# Simultaneous clustering of gene expression data with clinical chemistry and pathological evaluations reveals phenotypic prototypes

**DOI:** 10.1186/1752-0509-1-15

**Published:** 2007-02-23

**Authors:** Pierre R Bushel, Russell D Wolfinger, Greg Gibson

**Affiliations:** 1 National Center for Toxicogenomics, National Institute of Environmental Health Sciences, Research Triangle Park, North Carolina, USA; 2 SAS Institute, Cary, North Carolina, USA; 3 Department of Genetics, North Carolina State University, Raleigh, North Carolina, USA; 4 Bioinformatics Program, North Carolina State University, Raleigh, North Carolina, USA

## Abstract

**Background:**

Commonly employed clustering methods for analysis of gene expression data do not directly incorporate phenotypic data about the samples. Furthermore, clustering of samples with known phenotypes is typically performed in an informal fashion. The inability of clustering algorithms to incorporate biological data in the grouping process can limit proper interpretation of the data and its underlying biology.

**Results:**

We present a more formal approach, the mod*k*-prototypes algorithm, for clustering biological samples based on simultaneously considering microarray gene expression data and classes of known phenotypic variables such as clinical chemistry evaluations and histopathologic observations. The strategy involves constructing an objective function with the sum of the squared Euclidean distances for numeric microarray and clinical chemistry data and simple matching for histopathology categorical values in order to measure dissimilarity of the samples. Separate weighting terms are used for microarray, clinical chemistry and histopathology measurements to control the influence of each data domain on the clustering of the samples. The dynamic validity index for numeric data was modified with a category utility measure for determining the number of clusters in the data sets. A cluster's prototype, formed from the mean of the values for numeric features and the mode of the categorical values of all the samples in the group, is representative of the phenotype of the cluster members. The approach is shown to work well with a simulated mixed data set and two real data examples containing numeric and categorical data types. One from a heart disease study and another from acetaminophen (an analgesic) exposure in rat liver that causes centrilobular necrosis.

**Conclusion:**

The mod*k*-prototypes algorithm partitioned the simulated data into clusters with samples in their respective class group and the heart disease samples into two groups (sick and buff denoting samples having pain type representative of angina and non-angina respectively) with an accuracy of 79%. This is on par with, or better than, the assignment accuracy of the heart disease samples by several well-known and successful clustering algorithms. Following mod*k*-prototypes clustering of the acetaminophen-exposed samples, informative genes from the cluster prototypes were identified that are descriptive of, and phenotypically anchored to, levels of necrosis of the centrilobular region of the rat liver. The biological processes cell growth and/or maintenance, amine metabolism, and stress response were shown to discern between no and moderate levels of acetaminophen-induced centrilobular necrosis. The use of well-known and traditional measurements directly in the clustering provides some guarantee that the resulting clusters will be meaningfully interpretable.

## Background

Clustering of biological samples based on microarray gene expression data is now standard practice in clinical, biological, toxicological and pharmacological studies [1-4]. However, there are limitations to various clustering algorithms. For instance, the classic *k*-means clustering algorithm [5,6] uses Euclidean distance to measure dissimilarity and to cluster objects while the *k*-modes algorithm [7] only supports categorical or qualitative data through a simple matching objective function as a measure of dissimilarity. The inability of clustering algorithms to incorporate biological data in the grouping process can limit thorough interpretation of the data and its underlying biology.

Several approaches to incorporate biological data associated with samples into the analysis of gene expression data have been proposed recently. Shannon et al. [8] utilized Mantel statistics to correlate gene expression measurements with clinical covariates. The correlations are based on separate distance matrices computed using gene expression data and clinical covariates. Pearson correlation is used to assess main effects, whereas partial correlation coefficients are used to assess correlation between gene expression and a subset of the sample covariates conditioned on other sample covariates. Another approach introduced by Sese et al. [9] describes an itemset constrained clustering method where the optimal cluster that maximizes the interclass variance of gene expression with pathological features between groups is computed. Informative gene expression clusters annotated with disease descriptions of the liver were revealed. Kasturi and Acharya [10] proposed a model-free clustering method called information fusion, which uses SOMs Kohonen learning to update the weights for clusters and to essentially correlate microarray gene expression patterns with repeated motifs in the upstream region of genes. A potential limitation to this approach is that the grid of the nodes for the SOM has to be defined beforehand and the results of the clustering are dependent on the geometry of the grid. The development of additional methods to simultaneously cluster samples based on microarray gene expression data with associated biological information is reasonably expected to improve the grouping of samples and to enhance the discovery of biological processes that are correlated with phenotypic end-points.

Recent work has shown that better inference of genomic indicators for an outcome is obtained by integrating gene expression data with clinical or phenotypic data. For instance, Gevaert et al. [11] demonstrated that partial integration of clinical measurements with gene expression data through separate Bayesian networks that are joined by a single phenotype variable, improved the prediction of the prognosis of breast cancer. Others have used principal component analysis with an analysis of variance or partial least squares to associate gene expression data with clinical measurements for improved classification or prediction of an outcome [12,13]. In addition, a novel clustering approach that incorporates epigenetic (genes monitored for hypermethylation according to a binary [0,1] status) and phenotypic data (clinical measurements encoded as ordinal categorical variables), was shown to group tumor samples sufficiently well enough for discovery of informative pathways that adhere to strict heritability in breast cancer [14]. The approach, called heritable clustering, was suggested to be a framework to integrate other biological data. However, the extension of the algorithm for the analysis of high dimensional gene expression data integrated with clinical data as continuous measurements and phenotypic data as categorical values simultaneously has not been investigated.

Since the *k*-means and *k*-modes algorithms are efficient for processing large numeric and categorical data sets respectively, the combination of objective functions for measuring dissimilarity has been applied in the *k*-prototypes algorithm as a practical approach to extend the *k*-means-like algorithm for clustering large data sets with categorical values [7]. To test the utility of the *k*-prototypes algorithm for clustering samples based on numeric microarray gene expression data and clinical chemistry evaluations with histopathological observations as categorical values, we introduce a mod*k*-prototypes algorithm. The approach follows the *k*-means paradigm with randomization of initialization of the algorithm and is evaluated initially using two data sets. A simulated data set and a heart disease mixed type data set for proof-of-principle. The strategy involves constructing an objective function from the sum of the squared Euclidean distances for numeric data with simple matching for categorical values in order to measure dissimilarity of the samples. Separate weighting terms are used to control the influence of each data domain on the clustering of the samples. The dynamic validity index for numeric data was modified with a category utility measure in order to determine the optimal number of clusters in the mixed type data. A cluster's prototype is formed from the mean of the values for numeric features and the mode of the categorical values of all the samples in the group. The cluster's prototype is taken as a representation of the feature values that depicts the phenotype of the samples in the group.

Further rigorous investigation of the mod*k*-prototypes clustering method is then pursued by applying it to gene expression data and associated phenotypic evaluations from acetaminophen-exposed rat liver samples. Acetaminophen, which is an analgesic, causes centrilobular necrosis in the rat liver at high dose exposures. Using a chi-square test and GO annotations of selected genes which significantly distinguish differences between prototypes of clusters of the acetaminophen data set across all three data domains, phenotypic prototypes were obtained which were descriptive of, and anchored to, necrosis of the centrilobular region of the rat liver. This is an end-point manifested from high dose exposures of acetaminophen in the rat liver.

## Results

### Clustering mixed data types

**Figure 1:**
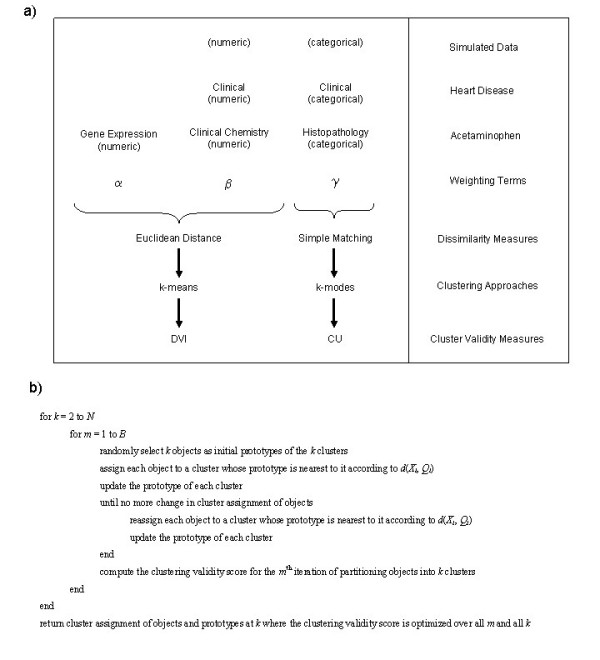
Modified *k*-prototypes clustering of mixed data types. a) The data sets used for clustering and the components of the mod*k*-prototypes algorithm. The type of the data is denoted in parentheses. b) The *k*-prototypes algorithm was modified (termed mod*k*-prototypes) to include *B *iterations of the assignment of the samples to the *k *number of clusters for each *k *= 2 to *N *number of samples. *d*(*X*_*i*_, *Q*_*l*_) is the dissimilarity function between the *i*^th ^sample and the *l*^th ^cluster prototype. The cluster prototypes are updated and the samples are reassigned repeatedly until there is no more change in cluster assignment. The validity score is computed for the final assignment of the samples. The number of clusters in the data is estimated by finding the assignment of the samples (over all *B *initializations and all *k *partitions) that yielded the optimal validity score.

The data sets used for clustering and the components of the mod*k*-prototypes algorithm are shown in Figure 1a. The *α*, *β *and *γ *weighting terms influence how much each data domain contributes to the clustering of the samples. An objective function with the sum of the squared Euclidean distances for numeric data and simple matching for categorical values is used to measure the dissimilarity of the samples. Samples are grouped using *k*-means clustering based on numeric attributes and *k*-modes clustering for attributes with categorical values. The DVI and CU measures comprise the DVI_CU score that measures the validity of the clustering. The mod*k*-prototypes algorithm is shown in Figure 1b and is a modification of the original *k*-prototypes algorithm [15]. For *k *= 2 to *N *number of samples and for *B *iterations, assignment of each sample is made to one of the *k *clusters based on the minimal distance of the sample to the prototypes of the clusters. The prototypes are updated and the samples are reassigned repeatedly until there is no more change in cluster assignment. The DVI_CU score is computed for the final assignment of the samples. The number of clusters in the data is estimated by finding the assignment of the samples, over all *B *initializations and all *k *partitions, which yielded the optimal validity score.

Initial validation of the mod*k*-prototypes algorithm was performed by evaluating the clustering of the samples in the simulated and the Cleveland Clinic heart disease mixed data sets. Clustering of the simulated data was performed with adaptive weighting of the numeric and categorical data. After 50 trial clustering attempts over 2 to *k *possible clusters in the data, the mod*k*-prototypes algorithm partitioned the data into 3 clusters with the samples in their respective class group (i.e., samples #s 12–22, 33–43 and 44–54 together respectively). Figure S3 in Additional file 1 illustrates the minimization of the DVI_CU index at *k *= 3.

**Figure 2:**
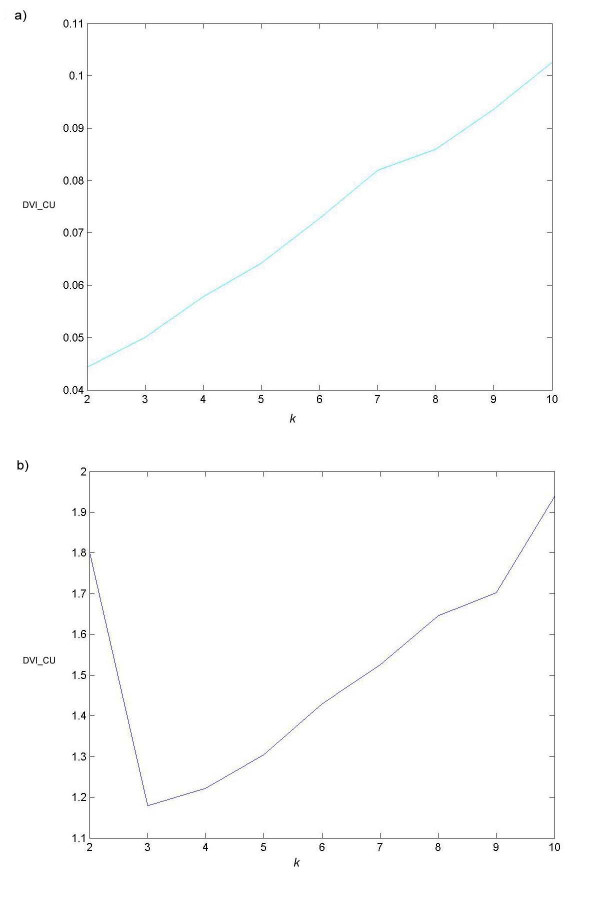
Determination of *k *clusters in the heart disease and acetaminophen data sets using mod*k*-prototypes. The a) heart disease data and b) acetaminophen data were clustered using the mod*k*-prototypes algorithm at values of *k *increasing from 2 to the *N *number of samples in the data. DVI_CU (on the y axis) was computed and plotted for the clustering of the data at each value of *k *(on the x axis). Only *k *= 2 to 10 is shown.

Clustering of the Cleveland Clinic heart disease data was performed with equal domain weighting. A plot of the DVI_CU validity measure at all values of *k *shows a minimum at *k *= 2, implying that the estimated number of clusters is two (Figure 2a). Additional file 2 shows the assignment of the samples to either of the two clusters along with the categorical value for the chest pain type attribute. Cluster 1 has 169 patient samples grouped together with pain type of angina suggestive of having heart disease (sick) while Cluster 2 has 134 patient samples grouped similarly together with non-angina representative of being without heart disease (buff). The accuracy of the clustering of the patients into the two groups was 79%. This is on par with, or better than, the classification accuracy of the samples by the NTGrowth, C4 and CLASSIT, and conceptual clustering classification algorithms which were reported to the University of California at Irvine repository for machine learning as 77%, 74.8% and 78.9% respectively. This analysis indicates that the mod*k*-prototypes algorithm can effectively cluster mixed data types leading to relatively accurate assignment of the samples to clusters with the appropriate clinical label.

Similarly, application of the mod*k*-prototypes algorithm with equal domain weighting to the acetaminophen mixed data indicates a minimum value for the DVI _CU validity measure at *k *= 3 (Figure 2b), implying that there are three clusters in the data. Ten samples were grouped into Cluster 1, nine into Cluster 2 and 45 into Cluster 3 (Additional file 3). The samples in Cluster 3 are comprised mostly of low dosed (50, 150 mg/kg) samples and high dosed (1500 and 2000 mg/kg) samples at 6 hrs except for 5 animals (rats #s 405, 406, 423, 518 and 520) that had low ALT and AST enzyme levels (Additional file 4). Elevated levels of ALT and AST correlate with liver injury. Cluster 2 contains all samples exposed to a high dose of acetaminophen for 18 or 24 hrs. Cluster 1 has samples exposed to high dose of acetaminophen for 48 hrs, except for moderate responder rats #s 407, 416 and 420, that were dosed for 18 or 24 hrs and had moderately elevated ALT and AST enzyme levels.

### Validation of clustering the acetaminophen mixed data

**Figure 3:**
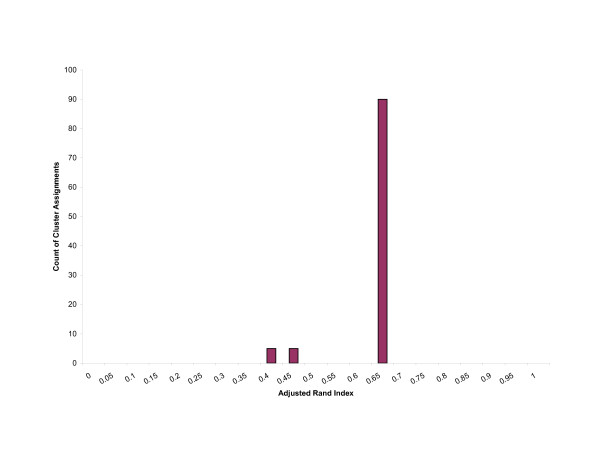
Validation of the cluster assignments for the acetaminophen data. mod*k*-prototypes clustering of the acetaminophen data was performed 100 times at *k *= 3 using equal weighting of the microarray, clinical chemistry and histopathology domain data. The necrosis of the centrilobular region of the rat liver histopathology observation was removed from the data prior to clustering and used as an external indicator of cluster assignment validation. The adjusted Rand index (x axis) was computed for each clustering of the data and graphed by count (y axis) of cluster assignments scored with the range of the index.

We next assessed the ability of the algorithm to cluster the samples according to the level of liver necrosis. At toxic doses of acetaminophen, glutathione is depleted leading to the formation of a reactive intermediate that covalently binds to sulfhydryl groups of several cellular proteins [16]. These adducts are thought to contribute to tissue necrosis [17]. The indicator variable representing the histopathological observations made by board-certified pathologists on the centrilobular region of the liver was removed from the data set prior to running the mod*k*-prototypes algorithm. This variable was then used as an external indicator to validate the assignment of samples to the three clusters. This observation has four feature values for all the exposed samples denoting either no, minimal, mild, or moderate severity of necrosis of the centrilobular region of the liver. Using the mod*k*-prototypes algorithm with *k *set at 3 and equal weighting of the microarray, clinical chemistry and histopathology domain data, 90% of the cluster assignments of the acetaminophen-treated samples had an adjusted Rand Index R' value greater than 0.64 when compared to the groups of the samples according to the observed level of necrosis (Figure 3). Since there were three clusters generated in the mixed data, yet four classes of acetaminophen-exposed centrilobular necrosis of the liver, perfect agreement was not possible, but the achieved clustering approached maximal validity given the external classification (Figures 2b and 3).

### Weighting schemes for clustering the acetaminophen mixed data

#### Proposed weighting of the domain data

Differential weighting of the domains may lead to further improved accuracy of the clustering procedure, as proposed by Lance and Williams [18] who introduced a clustering algorithm dependent on the weight of dissimilarity between objects [5]. User defined weights for clustering permit more or less influence to be given to particular components of the dissimilarity function. Several investigators at the NIEHS/National Center for Toxicogenomics responded to a survey in which they were asked to propose weights for clustering the acetaminophen microarray, clinical chemistry and histopathology data sets by assigning values for the mod*k*-prototypes algorithm parameters *α*, *β *and *γ *respectively. Their suggestions are listed in Table 1.

Two respondents, numbers 7 and 8, suggested different weighting schemes according to whether the end goal of the clustering of the samples was to either identify biomarkers related to histopathological changes following exposure to a toxicant, or to ascertain biological processes and pathways related to the phenotype of the samples. One respondent suggested two weighting schemes, one which is purported for data containing microarray, clinical chemistry and histopathology measurements (suggestion 5a) and one for molecular validation of toxicological evaluations of the samples (suggestion 5b). Two other respondents, numbers 14 and 15, suggested that the domain data be weighted according to preferred outcomes in the analysis of the data. The former proposed (i) equal weighting or (ii) coupling biology with phenotype whereas the latter proposed clustering based on (i) general effects of the treatment, (ii) specific injury and end-points from the exposure or (iii) the affected pathways. The averages of the suggested weights were 0.51, 0.24 and 0.25 for *α*, *β *and *γ *respectively. The standard deviations of the suggested weights were low (<0.16). However, the standard deviations of the *β *and *γ *weights were less than that of the *α *weight.

#### Simultaneous clustering using domain weights

Table 2 lists the determination of *k *and validation results of the simultaneous clustering of the acetaminophen microarray, clinical chemistry and histopathology data sets using the mod*k*-prototypes algorithm with specified weights. Equal weights of the domain data in the clustering process resulted in *k *= 3 and R' of 0.64 when the four centrilobular necrosis of the liver histopathology observation levels were used as an external indicator of clustering validity. Adaptive (*α*, *β *and *γ *adjusted to 0.26, 0.39 and 0.35 respectively) or proposed weighting of all three domain data yielded clustering results with *k *= 3, but R' = 0.64 or 0.67.

The highest agreement among all weighting schemes tested was achieved when the clinical chemistry data was given all of the weight. However, utilizing only the microarray data in the clustering process resulted in partitioning of the samples into just two groups with R' = 0.51. Excluding the microarray data from the analysis by weighting the clinical chemistry and the histopathology data equally yielded three clusters and R' = 0.64. Placing all the weight on the histopathology data or splitting the weighting equally between the microarray and histopathology data resulted in the poorest agreements (R' = 0.33 and 0.46 respectively) of the cluster assignments. Interestingly, having the weighting shared between at least the microarray and clinical chemistry domain data appears to be advantageous for clustering the data irrespective of the balancing of the weights. Surprisingly, no weighting scheme that included the histopathology data resulted in cluster groups with *k *> 4. However, partitions with *k *= 6 and *k *= 5 were obtained respectively, when clinical chemistry data alone and microarray with clinical chemistry data were used for clustering the samples. The latter resulted in R' = 0.66. With the weight of the microarray data set > 0.5 and some weight given to the histopathology data, weighting schemes for clustering of the biological samples validated with R' = 0.67 and *k *= 3 (the estimated number of clusters in the data [Figure 2b]).

### Phenotypic Prototypes

#### End-point components of the prototypes

The groups of samples from the mod*k*-prototypes algorithm were analyzed next for phenotypic prototypes by extracting histopathological feature value labels, clinical chemistry measurements, and genes from the prototypes of the clusters that (1) distinguish between pathologic outcomes and (2) best represent the underlying biology of the data. This analysis was performed on the acetaminophen microarray, clinical chemistry and histopathology data (including the centrilobular necrosis variable) with *k *= 3 and the *α*, *β *and *γ *weights set at 0.51, 0.24 and 0.25, respectively (see Tables 1 and 2 for the averages of the suggested weights). Table 3 lists partial prototypes of the resulting Clusters 1, 2, and 3 that represent samples grouped with moderate, no and mild levels, respectively, of necrosis of the centrilobular region. Samples in Clusters 1 and 3 were qualified by moderate and mild necrosis. By contrast, the majority of the samples in Cluster 2 were either low dosed (50 or 150 mg/kg) at any of the durations of exposure, or high dosed (1500 and 2000 mg/kg) for short durations (6 and 18 hrs). Except for 3 altered-responder rats (#s 423, 520 and 522) that were dosed for 24 or 48 hrs (Table 4). These exposures were expected to give at least a mild hepatotoxic phenotype. However, the ALT and AST levels for these animals were far below the treatment group average for these enzymes (see Additional file 4). Clusters 1 and 3 contained only high dosed samples (1500 and 2000 mg/kg) with the durations of exposure beyond 6 hrs (18, 24 or 48 hrs). In Cluster 3, most of the samples (6 of 9) were exposed for a time frame in which partial recovery from the treatment is expected (namely 48 hrs), whereas Cluster 1 only contains samples dosed for either 18 or 24 hrs. The samples in Clusters 1 and 3 also showed markedly and moderately elevated ALT and AST enzyme levels, as well as moderate and minimal congestion of the sinusoid region, respectively. Furthermore, the samples from the rats in Cluster 3 were represented by a histopathologic prototype characterized by minimal inflammatory cell infiltration in the centrilobular region, regeneration and degradation of the hepatocytes. The latter observed in the left medial lobe region. Samples from rats #s 407, 416 and 420 were dosed with 1500 mg/kg acetaminophen for either 18 or 24 hrs durations, but had only modest elevations of ALT and AST. Finally, from the prototype, samples in Cluster 1 were observed to have minimal hypertrophy of the hepatocytes predominantly. The rest of the histopathology feature values for the three clusters were not informative (all had no observed end-point) and therefore not included as representative features in the phenotypic prototypes.

Of the clinical chemistry measurements listed in Table 3 for each cluster prototype, ALT and AST levels clearly distinguish Cluster 1 samples labelled with the prototype feature as moderate necrosis of the centrilobular region of the liver from the two other clusters. In addition, elevated levels of TBA and decreased blood cholesterol differentiate samples in Cluster 1 from samples in Clusters 2 and 3 reasonably well.

#### Gene expression component of the prototypes

**Figure 4:**
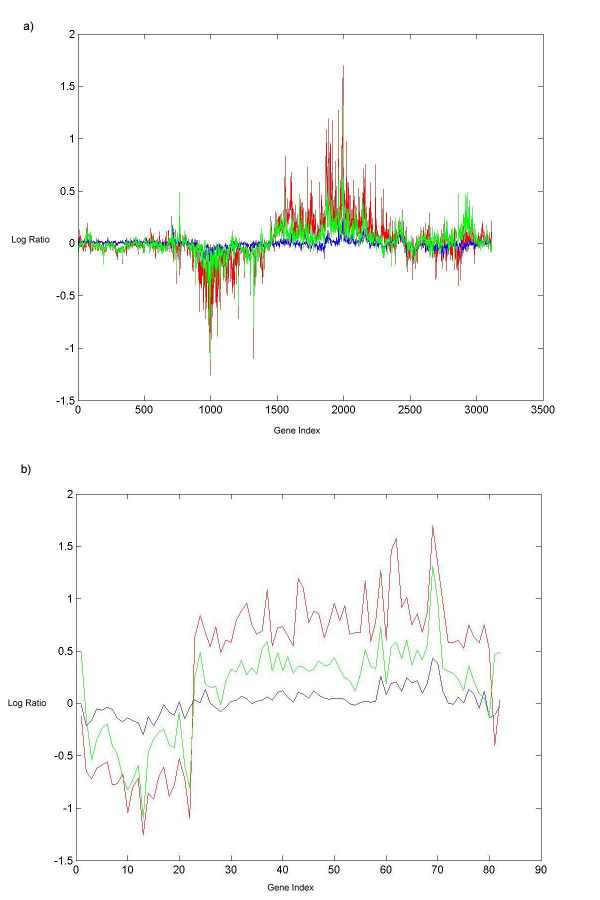
Gene expression components of the phenotypic prototypes. Plotting of the gene expression component of the prototypes from the clusters generated from clustering the acetaminophen data using the mod*k*-prototypes algorithm (with the levels of the necrosis of the centrilobular region of the rat liver included, 100 iterations and the average of the suggested weights of the domain data). a) All genes detected as significantly differentially expressed b) 82 genes significant and unique in distinguishing contrasts between the levels of necrosis of the centrilobular region of the rat liver. The red, blue and green lines denote the gene expression prototype from Clusters 1, 2 and 3 respectively. The log_10 _ratio values of the genes from the prototypes are signified on the y axis and the indices for the genes are denoted on the x axis.

Differences in gene expression levels between each cluster are shown in Figure 4a. The Cluster 2 prototype labelled with no necrosis of the centrilobular region had the least amount of differential gene expression of the samples in the cluster. Samples in Clusters 1 and 3 with moderate and mild necrosis of the centrilobular region as representative indicators respectively, had numerous genes with over 2-fold differential expression. The most dramatic gene expression differences were observed in the comparison of no versus moderate (Cluster 2 vs Cluster 1) necrosis of the centrilobular region of the liver, while the moderate versus mild comparison showed only slight differences in magnitude of expression between gene expression prototypes.

To extract genes from the pairwise comparisons of the expression component of the prototypes for the clusters that could statistically distinguish between levels of necrosis of the centrilobular region of the liver, a chi-square goodness-of-fit test was employed using the observed difference in a gene's expression ratios between two prototypes and the expected gene expression differences of all pairwise comparisons for all genes in the prototypes. With *α *set at 0.05, 82 genes, including several Cytochrome P450 genes and heme oxygenase 1, were identified as significant and unique in distinguishing contrasts between different levels of necrosis of the centrilobular region of the liver (Additional file 5). A subset of the genes is shown in Table 5. In particular, the GO biological processes cell growth and/or maintenance, amine metabolism and stress response discerned between clusters of samples grouped according to no and moderate necrosis of the centrilobular region of the rat liver. Mild and moderate centrilobular necrosis appeared to involve amine metabolism.

**Figure 5:**
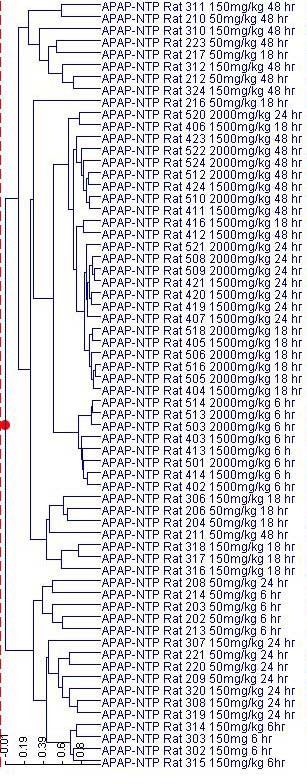
Hierarchical clustering of the biological samples. Log_10 _transformed gene expression ratio values of the 82 genes from the prototypes of the clusters of the biological samples were subjected to agglomerative hierarchical clustering using cosine correlation as the similarity measure and average linkage methodology. The branches of the dendrograms represent the amount of similarity between clusters of samples.

**Figure 6:**
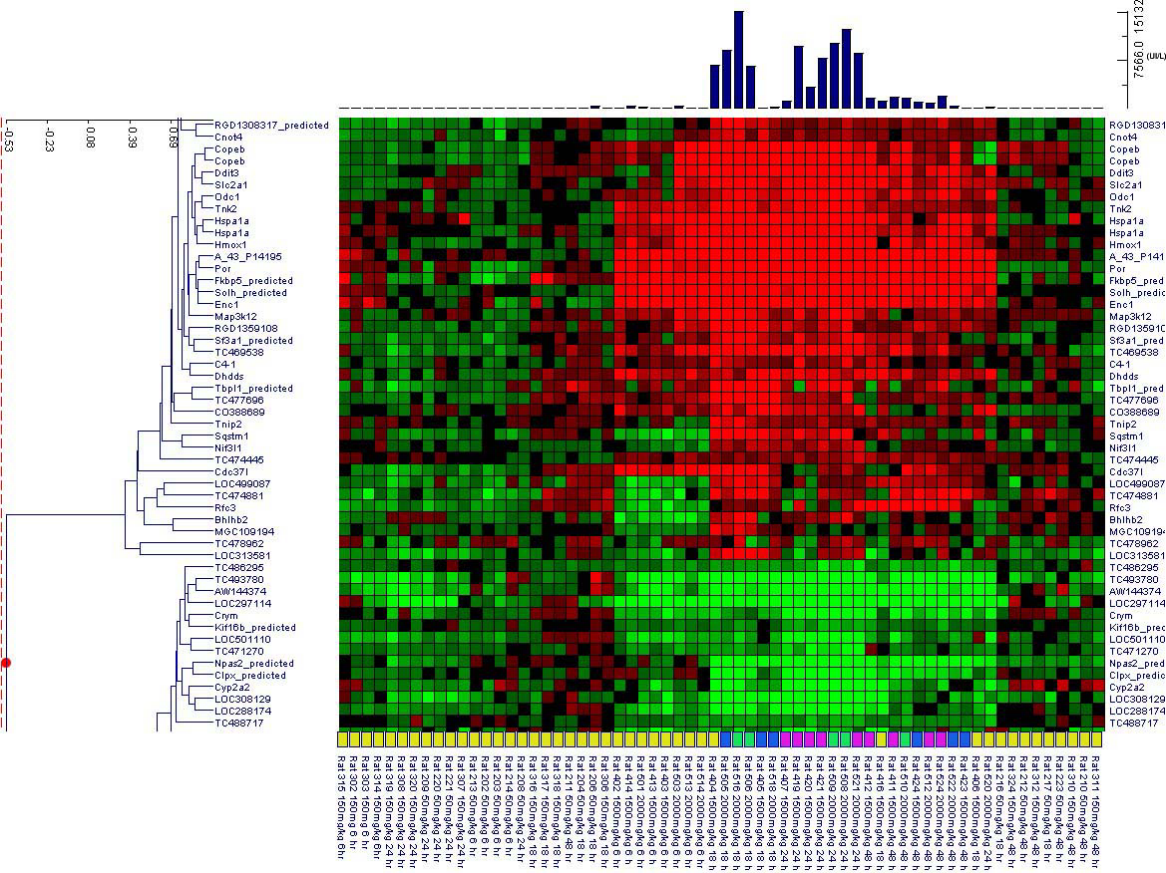
Two-way hierarchical clustering of the biological samples and the extracted genes. Log_10 _transformed gene expression ratio values of the 82 genes from the prototypes of the clusters of the biological samples were subjected to agglomerative hierarchical clustering as detailed in Figure 5. The resulting gene expression heat map contains the genes as the rows and samples as the columns with red indicating up regulation, green denoting down regulation and black signifying no change. At the top of the heat map, the level (UI/L) of ALT is plotted for each sample. At the bottom of the heat map, the severity of centrilobular necrosis observed is shown for each sample. (yellow, none; blue, minimal; magenta, mild; green, moderate).

A clearer picture of the differences between the samples in the clusters labelled with either no, mild or moderate necrosis of the centrilobular region of the rat liver was obtained by comparisons of Clusters 1, 2 and 3 using just the expression values of the 82 genes extracted from the prototypes (Figure 4b). About 75% of the genes progressively increase or decrease in differential expression as the level of necrosis of the centrilobular region of the liver transitions from no to mild to moderate. Finally, hierarchical clustering of the biological samples reveals very good grouping of the low dosed and high dosed samples. The latter very prominent and tight within time groups (Figure 5). Interestingly, as shown in Figure 6, the nine no- or moderately-responding rats (#s 405, 406, 407, 416, 420, 423, 518, 520 and 522) were distinctly different from their counterpart dose-by-time group subjects in terms of ALT enzyme levels. The high dosed 6 hrs rats differed from the high dosed 18, 24 and 48 hrs rats by a small cluster of genes that include an activator (Mitogen activated protein kinase kinase kinase 12 [Map3k12]) of the c-Jun N-terminal kinase (JNK) pathway, a transactivator of thyroid-stimulating hormone beta, and a regulator of neuronal differentiation and development.

## Discussion

Clustering of microarray gene expression data has matured by virtue of the growing number of analytical approaches for partitioning data. *k*-means is one of the most widely used unsupervised clustering methods for gene expression data. Unfortunately, *k*-means clustering, and other approaches such as SOMs do not guarantee globally optimal partitioning, require specifying the number of clusters or the configuration of the underlying classification structure, and suffer from inflexibility with respect to incorporation of associated biological data. More importantly, most clustering algorithms support only quantitative or qualitative data but not both simultaneously. Huang [15] introduced the *k*-prototypes algorithm that utilizes the clustering objective function of *k*-means for numeric measurements and *k*-modes for categorical values to partition data. We have proposed modifying this algorithm by adding an objective function to support and weight multi-domain, mixed type biological data within the *k*-means clustering paradigm. The advantage of our mod*k*-prototypes algorithm is that simultaneous clustering of gene expression data with clinical chemistry evaluations and histopathology observations results in informative clusters that are formed with prototypes of genes and values from end-point variables that are anchored to the phenotypes of samples with similar biological outcomes.

Our method is one of a class of approaches that seek to incorporate biological data directly into the clustering process [9,14]. Using necrosis of the centrilobular region of the rat liver following acetaminophen exposure as an end-point to couple with gene expression profiles, clinical chemistry evaluations and histopathological observations, simultaneous clustering of the data with the mod*k*-prototypes algorithm revealed phenotypic prototypes which were capable of distinguishing between no, mild and moderate levels of necrosis of the liver (Tables 3 to 5; Figure 4). For instance, non- or moderately-responding rats to acetaminophen exposure were distinctly different from their counterpart dose-by-time group subjects. Furthermore, the high dosed 6 hrs rats vs the high dosed 18, 24 and 48 hrs rats differed by a small cluster of genes involved in signal transduction and growth regulation. Not surprisingly, Cytochrome P450 genes and heme oxygenase 1, which have functions in detoxification and redox regulation in response to oxidative stress, were found to be indicators of toxicity in the gene expression component of the phenotypic prototypes that differentiated between levels of necrosis of the centrilobular region of the rat liver (Table 5). Several published reports of gene expression data generated from treatment of biological samples with toxic agents describe the altered expression of genes such as these in well-known biological pathways that are perturbed subsequent to incipient toxicity [19-24].

Weighting of the terms in the mod*k*-prototypes algorithm offers the flexibility to balance the influence of each domain of the data while simultaneously clustering the mixed data (see equation 1). This is advantageous for semi-supervised clustering when different goals for analyzing the data are in mind. The interest might be to cluster biological samples based on gene expression data with clinical chemistry measurements and histopathology observations for the purpose of finding biomarkers related to histopathological changes, or identifying which biological processes and pathways are related to the phenotypic end-point. From empirical analysis of acetaminophen-treated rat liver sample data using adaptive weighting or different weighting schemes, giving some weight to histopathology observations and at least half of the weight to the microarray data set is advantageous to clustering the data (Table 2). Interestingly, although applying all the weight to the clinical chemistry data gave the best fit between cluster assignment and histopathology evaluation of centrilobular necrosis, the number of clusters in the data was overestimated. This indicates that improper weighting of the domain data can potentially bias the clustering of the samples. Further work is being done to weight the domain data heuristically.

The high dimensionality of data has challenged the efficiency and reliability of clustering algorithms for quite sometime. In high dimensional space, data points become sparse making the use of some distance measures meaningless. However, results from experiments on real-world high dimensional data have shown that distance measures based on the Minkowski *L*_*d *_metrics, where *d *is either 1 or 2, increases or remains constant as the dimensionality of the data increases [25]. Our mod*k*-prototypes algorithm is based on the Euclidean (*L*_2_) distance metric for the high dimensional microarray data and clinical chemistry data. Given the aforementioned theoretical work plus our own simulation of a smaller scaled data set and reduction of the high dimensional numeric data (see Additional file 1), we are convinced that the clustering of the samples using the mod*k*-prototypes algorithm is not dependent on the scale or dimensionality of the data. The simulation results also provide evidence that the algorithm is at least able to find a small number of true/known clusters when they exist. Furthermore, the phenotypically anchored genes that were acquired from the prototypes of the clusters from the acetaminophen-exposed samples suggest that the mod*k*-prototypes algorithm forms groups of samples that are biologically meaningful. Additional applications of the method to a variety of simulated and real data sets are underway. These should also help in determining its usefulness over a range of scales and data dimensions.

As more biological data becomes available, sophisticated methods for clustering integrated data will be necessary in order to glean more meaningful information about the underlying biology of the samples. Efforts such as integrative genomics, systems biology, toxicogenomics, pharmacogenomics and biomedical informatics are generating volumes of biological data and information spanning transcriptomics, proteomics, metabolomics, toxicology, pharmacology, clinical biology and genetics to leverage each domain data for a more informed assessment of biological outcomes [12,26-30]. Case in point, the work of Baskin et al. [31] to collectively analyze microarray, clinical data and pathology observations revealed that gene expression patterns were very much consistent with the clinical outcomes, gross pathology and histopathology from influenza virus-infected pigtailed macaques primates. However, the identified clusters may not contain genes that are directly associated with the appearance of clinical signs or pathological indications of tissue infection because the domains of data were analyzed independently.

The mod*k*-prototypes algorithm is well-suited as a clustering method for grouping biological samples constrained by integrated data and feature values. It yields representatives of the clusters (the prototypes) which can potentially provide an initial insight to the biological mechanism driving the similarities of the samples and the phenotypes associated with gene expression. This concept of phenotypic anchoring has been proposed and tested as a means to link the cause of a disease or response with gene expression patterns and the altered biological processes that follow the observed effect [32-34]. We propose that the mod*k*-prototypes clustering method will provide a feasible computational alternative to embark on bridging multi-domain data analysis frameworks for integrative genomics, systems biology, pharmacology and toxicology.

## Conclusion

Many existing methods for clustering gene expression data do not incorporate phenotypic data about the samples. We developed the mod*k*-prototypes algorithm using an objective function with the sum of the squared Euclidean distances and simple matching for clustering biological samples based on numeric data and categorical values respectively. It is a formal approach to cluster gene expression data with phenotypic data. The algorithm is based on the original *k*-prototypes algorithm but is adapted along the *k*-means paradigm, it contains weighting terms for microarray, clinical and histopathology data, and is designed to determine the number of clusters in the data by minimizing a DVI_CU measure over all possible numbers of clusters and randomization of the initialization of the algorithm.

The advantage of simultaneous clustering of gene expression data with clinical chemistry evaluations and histopathology observations is that informative clusters are formed with prototypes of genes and end-point features that are linked to the phenotypes of samples with similar biological outcomes. Following mod*k*-prototypes clustering of the acetaminophen data with weighting of the domain data, informative genes from the cluster prototypes were identified that are descriptive of, and phenotypically anchored to, levels of necrosis of the centrilobular region of the rat liver. From empirical analysis of acetaminophen-treated rat liver sample data using adaptive weighting or different weighting schemes, having some weight given to the histopathology observations and weight of the microarray data set > 0.5, are advantageous to clustering the samples. Clustering the mixed data types in this fashion was better than typical *k*-means style clustering of either microarray or clinical chemistry numeric data alone (i.e. the other data sets weights set at 0) and better than *k*-modes clustering of the samples based solely on the histopathology data. We found that the expression profiles of several Cytochrome P450 genes and heme oxygenase 1 were significant in their differentiation between levels of centrilobular necrosis of the rat liver. Cytochrome P450 genes are in high proportion in the liver and produce detoxification enzymes to metabolize toxicants. Furthermore, the high dosed 6 hrs rats vs the high dosed 18, 24 and 48 hrs rats differed by a small cluster of genes containing an activator of the c-Jun N-terminal kinase pathway, a transactivator of thyroid-stimulating hormone beta and a regulator of neuronal differentiation and development. But overall, cell growth and/or maintenance, amine metabolism and stress response were biological processes that discerned between no and moderate levels of acetaminophen-induced necrosis of the centrilobular region of the rat liver. The use of well-known and traditional measurements directly in the clustering process provides some guarantee that the resulting clusters will be meaningfully interpretable. However, we realize that improper weights for the domain data can bias the clustering of the samples. In future work, we will investigate weighting the domain data heuristically.

## Methods

### Heart disease mixed data

Heart disease data from the Cleveland Clinic heart disease database maintained at the University of California at Irvine repository for machine learning was used as a data set with mixed features to evaluate the ability of the clustering algorithm to group samples based on mixed data types. The data set consists of 303 patients defined by 13 clinical features, five of which are numeric and eight categorical or nominal. The data has two classes: 165 individuals with no heart disease (buff) and 138 individuals with heart disease (sick).

### Acetaminophen microarray gene expression data and analysis

Microarray gene expression data was derived from left liver lobe mRNA samples collected from 4 male Fischer F344/N rats per dose group exposed to either 50 mg/kg, 150 mg/kg (low doses), 1500 mg/kg or 2000 mg/kg (high doses) body weight acetaminophen during a light period (between 12 noon and 1 pm) as well as liver mRNA collected from control (vehicle-treated) male rats [35]. Animals were sacrificed and mRNA extracted from liver specimens 6, 18, 24, or 48 hrs after treatment. Each RNA sample from a treated animal was compared with a pool of time-matched control mRNAs and analyzed in duplicate (dye reversal experiments) on Agilent-011868 (G4130A) rat oligonucleotide microarrays (Agilent Technologies, Palo Alto, CA). Acetaminophen exposure to the rat liver at 50 and 150 mg/kg is subtoxic. However, 1500 and 2000 mg/kg doses induce severe toxicity which peaks 24 hrs after exposure but the rats show signs of recovery 48 hrs after exposure.

Scanning of the microarray chips and acquisition of data from scanned images were as previously described [22]. Briefly, background subtracted pixel intensity values were log transformed, normalized and assessed for significance of expression (*p*-value < 0.05, Bonferroni corrected) using an ANOVA model comparing treated samples with time-matched controls. The approximately 3100 significant genes' pixel intensity ratio values from dye reversal hybridizations were combined (same subjects only) using Rosetta Resolver version 5.1.0.1.23 (Rosetta Biosoftware, Seattle, WA) error model weighted averaging [36,37]. Two gene features (A_43_P22641 and A_43_P22629), which had all values missing, were removed from analysis. The data used for clustering is in Additional file 6.

### Acetaminophen histopathology observations and clinical chemistry evaluations

48 histopathological observations of the acetaminophen-treated rat liver specimen slides and 10 clinical chemistry measurements on biosamples from the treated animals were collected as previously described [35]. Observations include: inflammatory cell infiltration of the centrilobular region or region not otherwise specified, necrosis of the centrilobular region or of hepatocytes, hyperplasia of the centrilobular hepatocytes, glycogen depletion, degeneration or regeneration of the hepatocytes or the centrilobular region, congestion or glycogen depletion of the centrilobular region or sinusoid and hyperplasia of the bile duct. Microscopic qualifiers were categorized as no, minimal, mild, moderate or marked. Discrepancies in histopathology observations were resolved by a team of board-certified pathologists [38].

Clinical chemistry evaluations of serum samples were performed using a Roche Cobas Fara chemistry analyzer (Roche Diagnostic Systems, Westwood, NJ) to numerically measure serum enzyme levels. These included indicators of liver injury (Alanine Aminotransferase [ALT] and Aspartate aminotransferase [AST]), Sorbitol dehydrogenase [SDH], cholesterol levels, indication of renal injury (urea nitrogen [BUN]), assessment of cholestasis – bile flow interruption (total bile acids [TBA], Creatine [Creat], Alkaline Phosphatase [ALP]), total protein (TP) and albumin (ALB) levels. Elevated levels of ALT and AST correlate with liver injury [39]. Missing values were imputed for rats #s 308 and 309 with either the group average or the overall mean value for each evaluation.

### Simulation of data for clustering using the mod*k*-prototypes algorithm

#### Numeric data

A data set comprised of numeric data with 64 features and 33 objects was simulated from three distinct probability distributions. Normal deviates (mean 0 and standard deviation 20) were drawn at random from the 3 probability distributions generating 11 objects in each. Samples that belong to the same class are #s12–22, 33–43, and 44–54 respectively.

#### Categorical data

A data set comprised of categorical data with 10 features and 33 objects was simulated from an HMM using R code in the HMM discrete non-parametric package. The HMM contained 3 states modelled on levels of toxicity (no/low, moderate and severe) and 5 severity levels (none, minimal, mild, moderate and marked) of centrilobular necrosis observed in rat livers exposed to acetaminophen. An independent cDNA microarray gene expression data set [22] acquired from rat liver samples exposed to 50, 150, 1500 and 2000 mg/kg of acetaminophen for 6, 24 and 48 hrs was used to estimate transition probabilities from a set of ~700 differentially expressed genes as well as a set of 700 genes selected at random. A third set of transition probabilities were manually created with a high probability (*p *>= 0.6) of visiting or remaining in the no/low toxicity state. An illustration of the HMM, a curve of the log-likelihood from the training, the transition and emission probabilities are in the supplemental materials (Additional file 1).

A mixed data set (Additional file 7) was created from the merge of the numeric and the categorical simulated data sets.

### Modified *k *(mod*k*)-prototypes algorithm

The Huang [15]*k*-prototypes algorithm which combines the *k*-means and the *k*-modes objective functions for clustering numeric data and categorical values respectively, was modified to follow the *k*-means algorithm paradigm, and was also optimized to search for clusters formed closest to the global minima of the objective function. In addition, a separate numeric objective function was utilized for the microarray and the clinical chemistry data resulting in the following mod*k*-prototypes objective function:

d(Xi,Ql)=α∑j=1mr(xijr−qljr)2+β∑j=1ms(xijs−qljs)2+γ∑j=1msδ(xijc,qljc)     (1)
 MathType@MTEF@5@5@+=feaafiart1ev1aaatCvAUfKttLearuWrP9MDH5MBPbIqV92AaeXatLxBI9gBaebbnrfifHhDYfgasaacH8akY=wiFfYdH8Gipec8Eeeu0xXdbba9frFj0=OqFfea0dXdd9vqai=hGuQ8kuc9pgc9s8qqaq=dirpe0xb9q8qiLsFr0=vr0=vr0dc8meaabaqaciaacaGaaeqabaqabeGadaaakeaacqWGKbazcqGGOaakcqWGybawdaWgaaWcbaGaemyAaKgabeaakiabcYcaSiabdgfarnaaBaaaleaacqWGSbaBaeqaaOGaeiykaKIaeyypa0dcciGae8xSde2aaabCaeaacqGGOaakcqWG4baEdaqhaaWcbaGaemyAaKMaemOAaOgabaGaemOCaihaaOGaeyOeI0IaemyCae3aa0baaSqaaiabdYgaSjabdQgaQbqaaiabdkhaYbaakiabcMcaPmaaCaaaleqabaGaeGOmaidaaaqaaiabdQgaQjabg2da9iabigdaXaqaaiabd2gaTnaaBaaameaacqWGYbGCaeqaaaqdcqGHris5aOGaey4kaSIae8NSdi2aaabCaeaacqGGOaakcqWG4baEdaqhaaWcbaGaemyAaKMaemOAaOgabaGaem4CamhaaOGaeyOeI0IaemyCae3aa0baaSqaaiabdYgaSjabdQgaQbqaaiabdohaZbaakiabcMcaPmaaCaaaleqabaGaeGOmaidaaaqaaiabdQgaQjabg2da9iabigdaXaqaaiabd2gaTnaaBaaameaacqWGZbWCaeqaaaqdcqGHris5aOGaey4kaSIae83SdC2aaabCaeaacqWF0oazcqGGOaakcqWG4baEdaqhaaWcbaGaemyAaKMaemOAaOgabaGaem4yamgaaOGaeiilaWIaemyCae3aa0baaSqaaiabdYgaSjabdQgaQbqaaiabdogaJbaakiabcMcaPaWcbaGaemOAaOMaeyypa0JaeGymaedabaGaemyBa02aaSbaaWqaaiabdohaZbqabaaaniabggHiLdGccaWLjaGaaCzcamaabmaabaacbeGae4xmaedacaGLOaGaayzkaaaaaa@89A4@

where *X*_*i *_is the *i*^th ^sample, for *i *= 1 to *N *number of samples, *Q*_*l *_is the *l*^th ^prototype, for *l *= 1 to *k *number of clusters, *m*_*r *_is the number of microarray numeric attributes, *m*_*s *_is the number of clinical chemistry numeric attributes, *m*_*c *_is the number of histopathological categorical attributes, *α*, *β *and *γ *denote the weights (*W*) for the microarray, clinical chemistry and histopathology data domain dissimilarity measures, respectively. The weights for data domain *d *at the *n*^th ^step (*W*_*d *_[*n*]) are adapted (for controlling how much each data domain contributes to the clustering of the samples) as follows:

Wd[n]={13n=0(1−τ)×Wd[n−1]+τ×avecorr(Xd,Qd)otherwise     (2)
 MathType@MTEF@5@5@+=feaafiart1ev1aaatCvAUfKttLearuWrP9MDH5MBPbIqV92AaeXatLxBI9gBaebbnrfifHhDYfgasaacH8akY=wiFfYdH8Gipec8Eeeu0xXdbba9frFj0=OqFfea0dXdd9vqai=hGuQ8kuc9pgc9s8qqaq=dirpe0xb9q8qiLsFr0=vr0=vr0dc8meaabaqaciaacaGaaeqabaqabeGadaaakeaacqWGxbWvdaWgaaWcbaGaemizaqgabeaakiabcUfaBjabd6gaUjabc2faDjabg2da9maaceqabaqbaeqabiGaaaqaamaaliaabaGaeGymaedabaGaeG4mamdaaaqaaiabd6gaUjabg2da9iabicdaWaqaaiabcIcaOiabigdaXiabgkHiTGGaciab=r8a0jabcMcaPiabgEna0kabdEfaxnaaBaaaleaacqWGKbazaeqaaOGaei4waSLaemOBa4MaeyOeI0IaeGymaeJaeiyxa0Laey4kaSIae8hXdqNaey41aqlcbaGae4xyaeMae4NDayNae4xzauMae43yamMae43Ba8Mae4NCaiNae4NCaiNaeiikaGIaemiwaG1aaWbaaSqabeaacqWGKbazaaGccqGGSaalcqWGrbqudaahaaWcbeqaaiabdsgaKbaakiabcMcaPaqaaiab+9gaVjab+rha0jab+HgaOjab+vgaLjab+jhaYjab+Dha3jab+LgaPjab+nhaZjab+vgaLbaaaiaawUhaaiaaxMaacaWLjaWaaeWaaeaaieqacqqFYaGmaiaawIcacaGLPaaaaaa@711F@

where tau (*τ*) is the exponential weighting update factor in the range [0,1] and avecorr(*X*^*d*^, *Q*^*d*^) is the average correlation coefficient (Pearson for numeric data, Jaccard for categorical data) between the samples and the prototypes based on the feature values from domain *d*.

avecorr(Xd,Qd)={(1N)∑i=1,Xid∈ClN(cov⁡(Xid,Qld)sXidsQld)2if domian d is numeric(1N)∑i=1,Xid∈ClN(p(Xid,Qld)p(Xid,Qld)+2q(Xid,Qld))if domian d is categorical
 MathType@MTEF@5@5@+=feaafiart1ev1aaatCvAUfKttLearuWrP9MDH5MBPbIqV92AaeXatLxBI9gBaebbnrfifHhDYfgasaacH8akY=wiFfYdH8Gipec8Eeeu0xXdbba9frFj0=OqFfea0dXdd9vqai=hGuQ8kuc9pgc9s8qqaq=dirpe0xb9q8qiLsFr0=vr0=vr0dc8meaabaqaciaacaGaaeqabaqabeGadaaakeaaieaacqWFHbqycqWF2bGDcqWFLbqzcqWFJbWycqWFVbWBcqWFYbGCcqWFYbGCcqGGOaakcqWGybawdaahaaWcbeqaaiabdsgaKbaakiabcYcaSiabdgfarnaaCaaaleqabaGaemizaqgaaOGaeiykaKIaeyypa0ZaaiqabeaafaqaaeGacaaabaWaaeWaaeaadaWccaqaaiabigdaXaqaaiabd6eaobaaaiaawIcacaGLPaaadaaeWbqaamaabmaabaWaaSGaaeaacyGGJbWycqGGVbWBcqGG2bGDcqGGOaakcqWGybawdaqhaaWcbaGaemyAaKgabaGaemizaqgaaOGaeiilaWIaemyuae1aa0baaSqaaiabdYgaSbqaaiabdsgaKbaakiabcMcaPaqaaiabdohaZnaaBaaaleaacqWGybawdaqhaaadbaGaemyAaKgabaGaemizaqgaaaWcbeaakiabdohaZnaaBaaaleaacqWGrbqudaqhaaadbaGaemiBaWgabaGaemizaqgaaaWcbeaaaaaakiaawIcacaGLPaaadaahaaWcbeqaaiabikdaYaaaaeaacqWGPbqAcqGH9aqpcqaIXaqmcqGGSaalcqWGybawdaqhaaadbaGaemyAaKgabaGaemizaqgaaSGaeyicI4Saem4qam0aaSbaaWqaaiabdYgaSbqabaaaleaacqWGobGta0GaeyyeIuoaaOqaaiab=LgaPjab=zgaMjabbccaGiabbsgaKjabb+gaVjabb2gaTjabbMgaPjabbggaHjabb6gaUjabbccaGGqaciab+rgaKjabbccaGiabbMgaPjabbohaZjabbccaGiabb6gaUjabbwha1jabb2gaTjabbwgaLjabbkhaYjabbMgaPjabbogaJbqaamaabmaabaWaaSGaaeaacqaIXaqmaeaacqWGobGtaaaacaGLOaGaayzkaaWaaabCaeaadaqadaqaamaaliaabaGaemiCaa3aaSbaaSqaaiabcIcaOiabdIfaynaaDaaameaacqWGPbqAaeaacqWGKbazaaWccqGGSaalcqWGrbqudaqhaaadbaGaemiBaWgabaGaemizaqgaaSGaeiykaKcabeaaaOqaaiabdchaWnaaBaaaleaacqGGOaakcqWGybawdaqhaaadbaGaemyAaKgabaGaemizaqgaaSGaeiilaWIaemyuae1aa0baaWqaaiabdYgaSbqaaiabdsgaKbaaliabcMcaPaqabaGccqGHRaWkcqaIYaGmcqWGXbqCdaWgaaWcbaGaeiikaGIaemiwaG1aa0baaWqaaiabdMgaPbqaaiabdsgaKbaaliabcYcaSiabdgfarnaaDaaameaacqWGSbaBaeaacqWGKbazaaWccqGGPaqkaeqaaaaaaOGaayjkaiaawMcaaaWcbaGaemyAaKMaeyypa0JaeGymaeJaeiilaWIaemiwaG1aa0baaWqaaiabdMgaPbqaaiabdsgaKbaaliabgIGiolabdoeadnaaBaaameaacqWGSbaBaeqaaaWcbaGaemOta4eaniabggHiLdaakeaacqWFPbqAcqWFMbGzcqqGGaaicqqGKbazcqqGVbWBcqqGTbqBcqqGPbqAcqqGHbqycqqGUbGBcqqGGaaicqGFKbazcqqGGaaicqqGPbqAcqqGZbWCcqqGGaaicqqGJbWycqqGHbqycqqG0baDcqqGLbqzcqqGNbWzcqqGVbWBcqqGYbGCcqqGPbqAcqqGJbWycqqGHbqycqqGSbaBaaaacaGL7baaaaa@E9AE@

where **cov **is the sample covariance, *s *is the sample standard deviation, *N *is the number of samples, *p *is the number of features that match and *q *is the number of features that do not match. The value of *τ *was set to 0.05 in order to adjust the weight of each domain by 5% at each iteration. The weights are non-negative and their sum is constrained to equal 1. The weights could potentially go to the boundaries [0,1] depending on the data. However, they can easily be constrained to always be above some lower bound, e.g. 0.05, or even fixed at proportions that are appropriate or reasonable to a domain expert.

Letting *z *represent *r *for microarray numeric data or *s *for clinical chemistry numeric data, the distance between Xiz
 MathType@MTEF@5@5@+=feaafiart1ev1aaatCvAUfKttLearuWrP9MDH5MBPbIqV92AaeXatLxBI9gBaebbnrfifHhDYfgasaacH8akY=wiFfYdH8Gipec8Eeeu0xXdbba9frFj0=OqFfea0dXdd9vqai=hGuQ8kuc9pgc9s8qqaq=dirpe0xb9q8qiLsFr0=vr0=vr0dc8meaabaqaciaacaGaaeqabaqabeGadaaakeaacqWGybawdaqhaaWcbaGaemyAaKgabaGaemOEaOhaaaaa@30EA@ and Qlz
 MathType@MTEF@5@5@+=feaafiart1ev1aaatCvAUfKttLearuWrP9MDH5MBPbIqV92AaeXatLxBI9gBaebbnrfifHhDYfgasaacH8akY=wiFfYdH8Gipec8Eeeu0xXdbba9frFj0=OqFfea0dXdd9vqai=hGuQ8kuc9pgc9s8qqaq=dirpe0xb9q8qiLsFr0=vr0=vr0dc8meaabaqaciaacaGaaeqabaqabeGadaaakeaacqWGrbqudaqhaaWcbaGaemiBaWgabaGaemOEaOhaaaaa@30E2@ containing missing values is defined as:

dj={0ifxijz or qljz is missingxijz−qljzotherwise.     (3)
 MathType@MTEF@5@5@+=feaafiart1ev1aaatCvAUfKttLearuWrP9MDH5MBPbIqV92AaeXatLxBI9gBaebbnrfifHhDYfgasaacH8akY=wiFfYdH8Gipec8Eeeu0xXdbba9frFj0=OqFfea0dXdd9vqai=hGuQ8kuc9pgc9s8qqaq=dirpe0xb9q8qiLsFr0=vr0=vr0dc8meaabaqaciaacaGaaeqabaqabeGadaaakeaacqWGKbazdaWgaaWcbaGaemOAaOgabeaakiabg2da9maaceqabaqbaeaabiGaaaqaaiabicdaWaqaaGqaaiab=LgaPjab=zgaMHqaciab+bcaGiab+Hha4naaDaaaleaacqGFPbqAcqGFQbGAaeaacqGF6bGEaaGccqqGGaaicqGFVbWBcqGFYbGCcqqGGaaicqWGXbqCdaqhaaWcbaGaemiBaWMaemOAaOgabaGaemOEaOhaaOGaeeiiaaIae8xAaKMae83CamNaeeiiaaIae8xBa0Mae8xAaKMae83CamNae83CamNae8xAaKMae8NBa4Mae83zaCgabaGaemiEaG3aa0baaSqaaiabdMgaPjabdQgaQbqaaiabdQha6baakiabgkHiTiabdghaXnaaDaaaleaacqWGSbaBcqWGQbGAaeaacqWG6bGEaaaakeaacqWFVbWBcqWF0baDcqWFObaAcqWFLbqzcqWFYbGCcqWF3bWDcqWFPbqAcqWFZbWCcqWFLbqzcqWFUaGlaaGaaCzcaiaaxMaadaqadaqaaGqabiab9ndaZaGaayjkaiaawMcaaaGaay5Eaaaaaa@71CC@

Then the distance between Xiz
 MathType@MTEF@5@5@+=feaafiart1ev1aaatCvAUfKttLearuWrP9MDH5MBPbIqV92AaeXatLxBI9gBaebbnrfifHhDYfgasaacH8akY=wiFfYdH8Gipec8Eeeu0xXdbba9frFj0=OqFfea0dXdd9vqai=hGuQ8kuc9pgc9s8qqaq=dirpe0xb9q8qiLsFr0=vr0=vr0dc8meaabaqaciaacaGaaeqabaqabeGadaaakeaacqWGybawdaqhaaWcbaGaemyAaKgabaGaemOEaOhaaaaa@30EA@ and Qlz
 MathType@MTEF@5@5@+=feaafiart1ev1aaatCvAUfKttLearuWrP9MDH5MBPbIqV92AaeXatLxBI9gBaebbnrfifHhDYfgasaacH8akY=wiFfYdH8Gipec8Eeeu0xXdbba9frFj0=OqFfea0dXdd9vqai=hGuQ8kuc9pgc9s8qqaq=dirpe0xb9q8qiLsFr0=vr0=vr0dc8meaabaqaciaacaGaaeqabaqabeGadaaakeaacqWGrbqudaqhaaWcbaGaemiBaWgabaGaemOEaOhaaaaa@30E2@ is:

d(Xiz,Qlz)=pp−p0∑j=1pdj2     (4)
 MathType@MTEF@5@5@+=feaafiart1ev1aaatCvAUfKttLearuWrP9MDH5MBPbIqV92AaeXatLxBI9gBaebbnrfifHhDYfgasaacH8akY=wiFfYdH8Gipec8Eeeu0xXdbba9frFj0=OqFfea0dXdd9vqai=hGuQ8kuc9pgc9s8qqaq=dirpe0xb9q8qiLsFr0=vr0=vr0dc8meaabaqaciaacaGaaeqabaqabeGadaaakeaacqWGKbazcqGGOaakcqWGybawdaqhaaWcbaGaemyAaKgabaGaemOEaOhaaOGaeiilaWIaemyuae1aa0baaSqaaiabdYgaSbqaaiabdQha6baakiabcMcaPiabg2da9maalaaabaGaemiCaahabaGaemiCaaNaeyOeI0IaemiCaa3aaSbaaSqaaiabicdaWaqabaaaaOWaaabCaeaacqWGKbazdaqhaaWcbaGaemOAaOgabaGaeGOmaidaaaqaaiabdQgaQjabg2da9iabigdaXaqaaiabdchaWbqdcqGHris5aOGaaCzcaiaaxMaadaqadaqaaGqabiab=rda0aGaayjkaiaawMcaaaaa@4F07@

where *d *is the Euclidean distance, *p *is the number of numeric features and *p*_0 _is the number of numeric features with missing values in Xiz
 MathType@MTEF@5@5@+=feaafiart1ev1aaatCvAUfKttLearuWrP9MDH5MBPbIqV92AaeXatLxBI9gBaebbnrfifHhDYfgasaacH8akY=wiFfYdH8Gipec8Eeeu0xXdbba9frFj0=OqFfea0dXdd9vqai=hGuQ8kuc9pgc9s8qqaq=dirpe0xb9q8qiLsFr0=vr0=vr0dc8meaabaqaciaacaGaaeqabaqabeGadaaakeaacqWGybawdaqhaaWcbaGaemyAaKgabaGaemOEaOhaaaaa@30EA@ and Qlz
 MathType@MTEF@5@5@+=feaafiart1ev1aaatCvAUfKttLearuWrP9MDH5MBPbIqV92AaeXatLxBI9gBaebbnrfifHhDYfgasaacH8akY=wiFfYdH8Gipec8Eeeu0xXdbba9frFj0=OqFfea0dXdd9vqai=hGuQ8kuc9pgc9s8qqaq=dirpe0xb9q8qiLsFr0=vr0=vr0dc8meaabaqaciaacaGaaeqabaqabeGadaaakeaacqWGrbqudaqhaaWcbaGaemiBaWgabaGaemOEaOhaaaaa@30E2@ or both.

For categorical (*c*) feature values, the dissimilarity measure between Xic
 MathType@MTEF@5@5@+=feaafiart1ev1aaatCvAUfKttLearuWrP9MDH5MBPbIqV92AaeXatLxBI9gBaebbnrfifHhDYfgasaacH8akY=wiFfYdH8Gipec8Eeeu0xXdbba9frFj0=OqFfea0dXdd9vqai=hGuQ8kuc9pgc9s8qqaq=dirpe0xb9q8qiLsFr0=vr0=vr0dc8meaabaqaciaacaGaaeqabaqabeGadaaakeaacqWGybawdaqhaaWcbaGaemyAaKgabaGaem4yamgaaaaa@30BC@ and Qlc
 MathType@MTEF@5@5@+=feaafiart1ev1aaatCvAUfKttLearuWrP9MDH5MBPbIqV92AaeXatLxBI9gBaebbnrfifHhDYfgasaacH8akY=wiFfYdH8Gipec8Eeeu0xXdbba9frFj0=OqFfea0dXdd9vqai=hGuQ8kuc9pgc9s8qqaq=dirpe0xb9q8qiLsFr0=vr0=vr0dc8meaabaqaciaacaGaaeqabaqabeGadaaakeaacqWGrbqudaqhaaWcbaGaemiBaWgabaGaem4yamgaaaaa@30B4@ is defined by the total number of mismatches of the corresponding histopathologic features from the sample and the prototype Xic
 MathType@MTEF@5@5@+=feaafiart1ev1aaatCvAUfKttLearuWrP9MDH5MBPbIqV92AaeXatLxBI9gBaebbnrfifHhDYfgasaacH8akY=wiFfYdH8Gipec8Eeeu0xXdbba9frFj0=OqFfea0dXdd9vqai=hGuQ8kuc9pgc9s8qqaq=dirpe0xb9q8qiLsFr0=vr0=vr0dc8meaabaqaciaacaGaaeqabaqabeGadaaakeaacqWGybawdaqhaaWcbaGaemyAaKgabaGaem4yamgaaaaa@30BC@ and Qlc
 MathType@MTEF@5@5@+=feaafiart1ev1aaatCvAUfKttLearuWrP9MDH5MBPbIqV92AaeXatLxBI9gBaebbnrfifHhDYfgasaacH8akY=wiFfYdH8Gipec8Eeeu0xXdbba9frFj0=OqFfea0dXdd9vqai=hGuQ8kuc9pgc9s8qqaq=dirpe0xb9q8qiLsFr0=vr0=vr0dc8meaabaqaciaacaGaaeqabaqabeGadaaakeaacqWGrbqudaqhaaWcbaGaemiBaWgabaGaem4yamgaaaaa@30B4@ respectively such that

d(Xic,Qlc)=∑j=1mcδ(xijc,qljc)     (5)
 MathType@MTEF@5@5@+=feaafiart1ev1aaatCvAUfKttLearuWrP9MDH5MBPbIqV92AaeXatLxBI9gBaebbnrfifHhDYfgasaacH8akY=wiFfYdH8Gipec8Eeeu0xXdbba9frFj0=OqFfea0dXdd9vqai=hGuQ8kuc9pgc9s8qqaq=dirpe0xb9q8qiLsFr0=vr0=vr0dc8meaabaqaciaacaGaaeqabaqabeGadaaakeaacqWGKbazcqGGOaakcqWGybawdaqhaaWcbaGaemyAaKgabaGaem4yamgaaOGaeiilaWIaemyuae1aa0baaSqaaiabdYgaSbqaaiabdogaJbaakiabcMcaPiabg2da9maaqahabaacciGae8hTdqMaeiikaGIaemiEaG3aa0baaSqaaiabdMgaPjabdQgaQbqaaiabdogaJbaakiabcYcaSiabdghaXnaaDaaaleaacqWGSbaBcqWGQbGAaeaacqWGJbWyaaGccqGGPaqkcaWLjaGaaCzcamaabmaabaacbeGae4xnaudacaGLOaGaayzkaaaaleaacqWGQbGAcqGH9aqpcqaIXaqmaeaacqWGTbqBdaWgaaadbaGaem4yamgabeaaa0GaeyyeIuoaaaa@559E@

where

δ(xijc,qljc)={0if xijc=qljc1if xijc≠qljc.     (6)
 MathType@MTEF@5@5@+=feaafiart1ev1aaatCvAUfKttLearuWrP9MDH5MBPbIqV92AaeXatLxBI9gBaebbnrfifHhDYfgasaacH8akY=wiFfYdH8Gipec8Eeeu0xXdbba9frFj0=OqFfea0dXdd9vqai=hGuQ8kuc9pgc9s8qqaq=dirpe0xb9q8qiLsFr0=vr0=vr0dc8meaabaqaciaacaGaaeqabaqabeGadaaakeaaiiGacqWF0oazcqGGOaakcqWG4baEdaqhaaWcbaGaemyAaKMaemOAaOgabaGaem4yamgaaOGaeiilaWIaemyCae3aa0baaSqaaiabdYgaSjabdQgaQbqaaiabdogaJbaakiabcMcaPiabg2da9maaceqabaqbaeaabiGaaaqaaiabicdaWaqaaGqaaiab+LgaPjab+zgaMjabbccaGiabdIha4naaDaaaleaacqWGPbqAcqWGQbGAaeaacqWGJbWyaaGccqGH9aqpcqWGXbqCdaqhaaWcbaGaemiBaWMaemOAaOgabaGaem4yamgaaaGcbaGaeGymaedabaGae4xAaKMae4NzayMaeeiiaaIaemiEaG3aa0baaSqaaiabdMgaPjabdQgaQbqaaiabdogaJbaakiabgcMi5kabdghaXnaaDaaaleaacqWGSbaBcqWGQbGAaeaacqWGJbWyaaGccqGGUaGlaaaacaGL7baacaWLjaGaaCzcamaabmaabaacbeGae0NnaydacaGLOaGaayzkaaaaaa@6584@

For *B *(typically set to 100) times, the mod*k*-prototypes algorithm initialization is seeded by the domain data vector of a randomly selected sample for each of the *k *clusters. For adaptive clustering, recursion was used to update the prototypes in order to find the configuration of the initial *k*-prototypes which ultimately results in the reduction of the objective function closest to the global minimum. Matlab code and a stand-alone executable program for the mod*k*-prototypes algorithm to simultaneously cluster gene expression data with clinical chemistry and pathological evaluations are available [48].

### Determination of cluster number (*k*) and validation of cluster assignment

To determine the number of clusters in a data set, the DVI of Shen et al. [40] was used. The DVI is based on an intra/inter ratio validity index that also includes scaling of the intra- and the inter-cluster distances.

DVIk={intra(k)max⁡i=2,...,N{intra(i)}+inter(k)max⁡i=2,...,N{inter(i)}}
 MathType@MTEF@5@5@+=feaafiart1ev1aaatCvAUfKttLearuWrP9MDH5MBPbIqV92AaeXatLxBI9gBaebbnrfifHhDYfgasaacH8akY=wiFfYdH8Gipec8Eeeu0xXdbba9frFj0=OqFfea0dXdd9vqai=hGuQ8kuc9pgc9s8qqaq=dirpe0xb9q8qiLsFr0=vr0=vr0dc8meaabaqaciaacaGaaeqabaqabeGadaaakeaaieaacqWFebarcqWFwbGvcqWFjbqsdaWgaaWcbaGae83AaSgabeaakiabg2da9maacmqabaWaaSaaaeaacqWFPbqAcqWFUbGBcqWF0baDcqWFYbGCcqWFHbqycqGGOaakcqWGRbWAcqGGPaqkaeaadaWfqaqaaiGbc2gaTjabcggaHjabcIha4bWcbaGaemyAaKMaeyypa0JaeGOmaiJaeiilaWIaeiOla4IaeiOla4IaeiOla4IaeiilaWIaemOta4eabeaakiabcUha7jab=LgaPjab=5gaUjab=rha0jab=jhaYjab=fgaHjabcIcaOiabdMgaPjabcMcaPiabc2ha9baacqGHRaWkdaWcaaqaaiab=LgaPjab=5gaUjab=rha0jab=vgaLjab=jhaYjabcIcaOiabdUgaRjabcMcaPaqaamaaxababaGagiyBa0MaeiyyaeMaeiiEaGhaleaacqWGPbqAcqGH9aqpcqaIYaGmcqGGSaalcqGGUaGlcqGGUaGlcqGGUaGlcqGGSaalcqWGobGtaeqaaOGaei4EaSNae8xAaKMae8NBa4Mae8hDaqNae8xzauMae8NCaiNaeiikaGIaemyAaKMaeiykaKIaeiyFa0haaaGaay5Eaiaaw2haaaaa@7E0F@

where

inter(k)=Maxi,j(‖Qi−Qj‖2)Mini≠j(‖Qi−Qj‖2)∑i=1k(1∑j=1k(‖Qi−Qj‖2)),
 MathType@MTEF@5@5@+=feaafiart1ev1aaatCvAUfKttLearuWrP9MDH5MBPbIqV92AaeXatLxBI9gBaebbnrfifHhDYfgasaacH8akY=wiFfYdH8Gipec8Eeeu0xXdbba9frFj0=OqFfea0dXdd9vqai=hGuQ8kuc9pgc9s8qqaq=dirpe0xb9q8qiLsFr0=vr0=vr0dc8meaabaqaciaacaGaaeqabaqabeGadaaakeaaieaacqWFPbqAcqWFUbGBcqWF0baDcqWFLbqzcqWFYbGCcqGGOaakcqWGRbWAcqGGPaqkcqGH9aqpdaWcaaqaamaaxababaGaemyta0KaemyyaeMaemiEaGhaleaacqWGPbqAcqGGSaalcqWGQbGAaeqaaOWaaeWaaeaadaqbdaqaaiabdgfarnaaBaaaleaacqWGPbqAaeqaaOGaeyOeI0Iaemyuae1aaSbaaSqaaiabdQgaQbqabaaakiaawMa7caGLkWoadaahaaWcbeqaaiabikdaYaaaaOGaayjkaiaawMcaaaqaamaaxababaGaemyta0KaemyAaKMaemOBa4galeaacqWGPbqAcqGHGjsUcqWGQbGAaeqaaOWaaeWaaeaadaqbdaqaaiabdgfarnaaBaaaleaacqWGPbqAaeqaaOGaeyOeI0Iaemyuae1aaSbaaSqaaiabdQgaQbqabaaakiaawMa7caGLkWoadaahaaWcbeqaaiabikdaYaaaaOGaayjkaiaawMcaaaaadaaeWbqaamaabmaabaWaaSaaaeaacqaIXaqmaeaadaaeWbqaamaabmaabaWaauWaaeaacqWGrbqudaWgaaWcbaGaemyAaKgabeaakiabgkHiTiabdgfarnaaBaaaleaacqWGQbGAaeqaaaGccaGLjWUaayPcSdWaaWbaaSqabeaacqaIYaGmaaaakiaawIcacaGLPaaaaSqaaiabdQgaQjabg2da9iabigdaXaqaaiabdUgaRbqdcqGHris5aaaaaOGaayjkaiaawMcaaaWcbaGaemyAaKMaeyypa0JaeGymaedabaGaem4AaSganiabggHiLdGccqGGSaalaaa@7E43@

*k *is the number of clusters, *N *is the number of samples and intra is the average Euclidean distance between samples and the prototype *Q *of the cluster each sample is assigned to.

For mixed data with numeric and categorical values, the DVI was modified to include a CU measure [41] that defines the probability of matching a categorical feature value given a cluster versus the probability of the categorical feature value given the entire data set

CUm=1m∑k=1mP(Ck)[∑i∑jP(Ai=Vij|Ck)2−∑i∑jP(Ai=Vij)2]     (7)
 MathType@MTEF@5@5@+=feaafiart1ev1aaatCvAUfKttLearuWrP9MDH5MBPbIqV92AaeXatLxBI9gBaebbnrfifHhDYfgasaacH8akY=wiFfYdH8Gipec8Eeeu0xXdbba9frFj0=OqFfea0dXdd9vqai=hGuQ8kuc9pgc9s8qqaq=dirpe0xb9q8qiLsFr0=vr0=vr0dc8meaabaqaciaacaGaaeqabaqabeGadaaakeaacqWGdbWqcqWGvbqvdaWgaaWcbaGaemyBa0gabeaakiabg2da9maaliaabaGaeGymaedabaGaemyBa0gaamaaqahabaGaemiuaaLaeiikaGIaem4qam0aaSbaaSqaaiabdUgaRbqabaGccqGGPaqkaSqaaiabdUgaRjabg2da9iabigdaXaqaaiabd2gaTbqdcqGHris5aOWaamWaaeaadaaeqbqaamaaqafabaGaemiuaaLaeiikaGIaemyqae0aaSbaaSqaaiabdMgaPbqabaGccqGH9aqpcqWGwbGvdaWgaaWcbaGaemyAaKMaemOAaOgabeaakiabcYha8jabdoeadnaaBaaaleaacqWGRbWAaeqaaOGaeiykaKYaaWbaaSqabeaacqaIYaGmaaaabaGaemOAaOgabeqdcqGHris5aaWcbaGaemyAaKgabeqdcqGHris5aOGaeyOeI0YaaabuaeaadaaeqbqaaiabdcfaqjabcIcaOiabdgeabnaaBaaaleaacqWGPbqAaeqaaOGaeyypa0JaemOvay1aaSbaaSqaaiabdMgaPjabdQgaQbqabaGccqGGPaqkdaahaaWcbeqaaiabikdaYaaaaeaacqWGQbGAaeqaniabggHiLdaaleaacqWGPbqAaeqaniabggHiLdaakiaawUfacaGLDbaacaWLjaGaaCzcamaabmaabaacbeGae83naCdacaGLOaGaayzkaaaaaa@70BA@

where *P*(*A*_*i *_= *V*_*ij*_) is the unconditional probability of feature *A*_*i *_taking on the value *V*_*ij*_, *P*(*A*_*i *_= *V*_*ij *_| *C*_*k*_) is the conditional probability of *A*_*i *_= *V*_*ij *_given cluster *C*_*k*_, and *k *is the cluster number from 1 to *m*. The DVI modified with CU

DVI_CU = (DVI + 1/CU)     **(8)**

is minimized over all *k *sets for each run of the mod*k*-prototypes clustering algorithm. Validation of cluster assignment was carried out using R', the adjusted Rand index [42-44]. When two partitions agree totally, R' is 1 and when the partitions are selected by chance, R' is 0.

### Generation of phenotypic prototypes

A cluster's prototype is formed from the mean of the values for numeric features and the mode of the categorical values of all the samples in the group. Hence, the cluster's prototype is taken as a representation of the feature values that depicts the phenotype of the samples in the group. The process for obtaining phenotypic prototypes is to extract all the histopathologic feature value labels and clinical chemistry measurements as well as significant genes from the prototypes of the clusters that can distinguish between pathological outcomes and best represent the underlying biology of the group of samples. Let the observed difference between the expression ratio of the *g*th gene (*p *in total) from the gene expression component of prototype *q *for *i*th and *j*th (*i *not equal to *j*) cluster (*k *in total) be observed_*g *_= (*q*_*gi *_- *q*_*gj*_) and the expected change in expression be

expected=∑g=1p∑i=1k−1∑j=i+1k(qgik−qgjk)(k2)p
 MathType@MTEF@5@5@+=feaafiart1ev1aaatCvAUfKttLearuWrP9MDH5MBPbIqV92AaeXatLxBI9gBaebbnrfifHhDYfgasaacH8akY=wiFfYdH8Gipec8Eeeu0xXdbba9frFj0=OqFfea0dXdd9vqai=hGuQ8kuc9pgc9s8qqaq=dirpe0xb9q8qiLsFr0=vr0=vr0dc8meaabaqaciaacaGaaeqabaqabeGadaaakeaaieaacqWFLbqzcqWF4baEcqWFWbaCcqWFLbqzcqWFJbWycqWF0baDcqWFLbqzcqWFKbazcqGH9aqpdaWcaaqaamaaqahabaWaaabCaeaadaaeWbqaaiabcIcaOiabdghaXnaaBaaaleaacqWGNbWzcqWGPbqAcqWGRbWAaeqaaOGaeyOeI0IaemyCae3aaSbaaSqaaiabdEgaNjabdQgaQjabdUgaRbqabaGccqGGPaqkaSqaaiabdQgaQjabg2da9iabdMgaPjabgUcaRiabigdaXaqaaiabdUgaRbqdcqGHris5aaWcbaGaemyAaKMaeyypa0JaeGymaedabaGaem4AaSMaeyOeI0IaeGymaedaniabggHiLdaaleaacqWGNbWzcqGH9aqpcqaIXaqmaeaacqWGWbaCa0GaeyyeIuoaaOqaamaabmaabaqbaeqabiqaaaqaaiabdUgaRbqaaiabikdaYaaaaiaawIcacaGLPaaacqWGWbaCaaaaaa@64EE@

Averaging over all the genes gives an estimate of the expected difference between a gene's ratio values in the prototypes of two clusters being compared. Assuming independence and an approximately normal distribution of differences, genes which have expression ratios which significantly distinguish between prototypes of clusters are evaluated using a standard chi-square (*X*^2^) goodness-of-fit test [45]:

χc2=2(observedg−expected)2expected≥χα,12     (9)
 MathType@MTEF@5@5@+=feaafiart1ev1aaatCvAUfKttLearuWrP9MDH5MBPbIqV92AaeXatLxBI9gBaebbnrfifHhDYfgasaacH8akY=wiFfYdH8Gipec8Eeeu0xXdbba9frFj0=OqFfea0dXdd9vqai=hGuQ8kuc9pgc9s8qqaq=dirpe0xb9q8qiLsFr0=vr0=vr0dc8meaabaqaciaacaGaaeqabaqabeGadaaakeaaiiGacqWFhpWydaqhaaWcbaGaem4yamgabaGaeGOmaidaaOGaeyypa0ZaaSaaaeaacqaIYaGmcqGGOaakieaacqGFVbWBcqGFIbGycqGFZbWCcqGFLbqzcqGFYbGCcqGF2bGDcqGFLbqzcqGFKbazdaWgaaWcbaGaem4zaCgabeaakiabgkHiTiab+vgaLjab+Hha4jab+bhaWjab+vgaLjab+ngaJjab+rha0jab+vgaLjab+rgaKjabcMcaPmaaCaaaleqabaGaeGOmaidaaaGcbaGae4xzauMae4hEaGNae4hCaaNae4xzauMae43yamMae4hDaqNae4xzauMae4hzaqgaaiabgwMiZkab=D8aJnaaDaaaleaacqWFXoqycqGGSaalcqaIXaqmaeaacqaIYaGmaaGccaWLjaGaaCzcamaabmaabaacbeGae0xoaKdacaGLOaGaayzkaaaaaa@647B@

where the null hypothesis is that the expression value of the *g*^th ^gene does not distinguish between prototypes of a pair of clusters that are compared. The null hypothesis is rejected at a level of *α*, the probability of a type I error, if χc2
 MathType@MTEF@5@5@+=feaafiart1ev1aaatCvAUfKttLearuWrP9MDH5MBPbIqV92AaeXatLxBI9gBaebbnrfifHhDYfgasaacH8akY=wiFfYdH8Gipec8Eeeu0xXdbba9frFj0=OqFfea0dXdd9vqai=hGuQ8kuc9pgc9s8qqaq=dirpe0xb9q8qiLsFr0=vr0=vr0dc8meaabaqaciaacaGaaeqabaqabeGadaaakeaacqaHhpWydaqhaaWcbaGaem4yamgabaGaeGOmaidaaaaa@30D1@ ≥ *χ*^2^(1, *α*) where *χ*^2^(1, *α*) is the *α*-level critical value of a *χ*^2^-distribution with 1 degree of freedom. An *α *of 0.05 gives reliable results. Genes from a comparison of two prototypes which significantly distinguish the clusters are annotated for biological function and process(es) using the GO database [46,47].

## List of abbreviations used

mod*k*-prototypes, modified *k*-prototypes; ALT, Alanine aminotransferase; AST, Aspartate aminotransferase; SDH, Sorbitol dehydrogenase; BUN, blood urea nitrogen; TBA, total bile acids; Creat, Creatine; ALP, Alkaline Phosphatase; TP, total protein; ALB, albumin; DVI, dynamic validity index; CU, category utility; R', Adjusted Rand Index; DVI_CU, dynamic validity index with category utility; SOM, self organizing map; HMM, hidden Markov model; GO, Gene Ontology.

## Competing interests

The author(s) declare that they have no competing interests.

## Authors' contributions

PRB performed the analysis of the gene expression data, considered the utilization of the DVI_CU measure and the *k*-prototypes algorithm for gene expression and phenotypic data, implemented the mod*k*-prototypes clustering algorithm and DVI_CU, applied them to the mixed type data and wrote the paper. RDW provided statistical guidance for the work. GG provided advice and guidance throughout the project. Both GG and RDW assisted in the evaluation of results. All authors read and approved the final manuscript.

## Supplementary Material

Additional file 1 **Generation and clustering of simulated mixed data and real data with reduced dimensions of the microarray data**. Supplemental_materials.pdf is a pdf file to be viewed with Adobe Acrobat. Click here for file

Additional file 2 **Cluster assignment of the heart disease samples using equal weights**. Table_S1.txt is a tab-delimited text file to be opened and viewed with any standard spreadsheet software. Click here for file

Additional file 3 **Cluster assignment of the acetaminophen-treated samples using equal weights**. Table_S2.txt is a tab-delimited text file to be opened and viewed with any standard spreadsheet software. Click here for file

Additional file 4 **Histopathology observations and clinical chemistry measurements**. Table_S3.txt is a tab-delimited text file to be opened and viewed with any standard spreadsheet software. Click here for file

Additional file 5 **Significant and unique genes that distinguish between levels of centrilobular necrosis of the rat liver**. Table_S4.txt is a tab-delimited text file to be opened and viewed with any standard spreadsheet software. Click here for file

Additional file 6 **Approximately 3100 genes determined to be significantly differentially expressed by ANOVA modelling**. DEGs.txt is a tab-delimited text file to be opened and viewed with any standard spreadsheet software. Click here for file

Additional file 7 **Simulated mixed data of different types (numeric and categorical)**. sim_mixed_data.txt is a tab-delimited text file to be opened and viewed with any standard spreadsheet software. Click here for file

## Figures and Tables

**Table 1:** Proposed weighting schemes for the domain data.

**Expert**	**α**	**β**	**γ**
1	0.6	0.2	0.2
2	0.5	0.2	0.3
3	0.5	0.25	0.25
4	0.6	0	0.4
5a	0.2	0.5	0.3
5b	0.8	0.1	0.1
6	0.6	0.1	0.3
7a	0.4	0.4	0.2
7b	0.4	0.2	0.4
8a	0.6	0.3	0.1
8b	0.4	0.2	0.4
9	0.6	0.2	0.2
10	0.6	0.2	0.2
11	0.6	0.2	0.2
12	0.4	0.3	0.3
13	0.333	0.333	0.333
14a	0.333	0.333	0.333
14b	0.5	0.2	0.3
15a	0.7	0.2	0.1
15b	0.3	0.4	0.3
15c	0.7	0.2	0.1
Average	0.508	0.239	0.253
Standard Dev.	0.152	0.113	0.100

**Table 2:** Validation of clustering the samples from the acetaminophen data using mod*k*-prototypes.

**Weighting Scheme**	***α***	***β***	***γ***	**Adjusted Rand Index**	***k***
1	*0.333*	*0.333*	*0.333*	0.64	*3*
2	1	0	0	0.51	2
3	0	1	0	0.69	6
4	0	0	1	0.33	2
5	0.5	0.5	0	0.66	5
6	0.5	0	0.5	0.46	4
7	0	0.5	0.5	0.64	3
8	0.51	0.24	0.25	0.67	3
9	0.4	0.4	0.2	0.64	3
10	0.4	0.2	0.4	0.67	3
11	0.6	0.2	0.2	0.67	3
12	0.2	0.4	0.4	0.64	3
13*	0.26	0.39	0.35	0.64	3
14	0.8	0.1	0.1	0.67	3
15	0.7	0.15	0.15	0.67	3

*α*, *β *and *γ *denote the weights for the microarray, clinical chemistry and histopathology data domain dissimilarity measures, respectively. *k *is the number of clusters formed.

**Table 3:** Partial end-point components of the phenotypic prototypes from the clustering of the acetaminophen-treated samples.

	Cluster		
Features	1	2	3

Cong_Sinusoid	Moderate	None	Minimal
Necr_Cent	Moderate	None	Mild
Infl_Cent	None	None	Minimal/Mild*
Hypert_Hepa	Minimal	None	None
Regen_Hepa	None	None	Minimal
Dege_Hepa*	None	None	Minimal
ALB (g/dL)	5.16	5.03	4.78
ALP (IU/L)	413.22	323.43	368.78
ALT (IU/L)	9649.40	118.48	1676.10
AST (IU/L)	20304.00	171.80	2820.20
Creat (mg/dL)	0.70	0.70	0.70
BUN (mg/dL)	23.56	15.56	18.11
CHOLE (mg/dL)	59.78	86.54	85.44
TBA (umol/L)	61.67	7.61	43.56
SDH (IU/L)	2.89	32.41	398.89
TP (g/dL)	7.53	7.52	7.19
* Observed in the left medial lobe			

**Table 4:** Cluster assignment of the acetaminophen-treated samples.

Cluster 1		Cluster 2		Cluster 3	
Treatment	Animal #	Treatment	Animal #	Treatment	Animal #

1500 MG/K18 HR	404	50 MG/KG6 HR	202	1500 MG/K24 HR	407
1500 MG/K24 HR	419	50 MG/KG6 HR	203	1500 MG/K48 HR	411
1500 MG/K24 HR	421	50 MG/KG18 HR	204	1500 MG/K48 HR	412
2000 MG/K18 HR	505	50 MG/KG18 HR	206	1500 MG/K18 HR	416
2000 MG/K18 HR	506	50 MG/KG24 HR	208	1500 MG/K24 HR	420
2000 MG/K24 HR	508	50 MG/KG24 HR	209	1500 MG/K48 HR	424
2000 MG/K24 HR	509	50 MG/KG48 HR	210	2000 MG/K48 HR	510
2000 MG/K18 HR	516	50 MG/KG48 HR	211	2000 MG/K48 HR	512
2000 MG/K24 HR	521	50 MG/KG48 HR	212	2000 MG/K48 HR	524
		50 MG/KG6 HR	213		
		50 MG/KG6 HR	214		
		50 MG/KG18 HR	216		
		50 MG/KG18 HR	217		
		50 MG/KG24 HR	220		
		50 MG/KG24 HR	221		
		50 MG/KG48 HR	223		
		150 MG/KG6 HR	302		
		150 MG/KG6 HR	303		
		150 MG/KG18 HR	306		
		150 MG/KG24 HR	307		
		150 MG/KG24 HR	308		
		150 MG/KG48 HR	310		
		150 MG/KG48 HR	311		
		150 MG/KG48 HR	312		
		150 MG/KG6 HR	314		
		150 MG/KG6 HR	315		
		150 MG/KG18 HR	316		
		150 MG/KG18 HR	317		
		150 MG/KG18 HR	318		
		150 MG/KG24 HR	319		
		150 MG/KG24 HR	320		
		150 MG/KG48 HR	324		
		1500 MG/K6 HR	402		
		1500 MG/K6 HR	403		
		1500 MG/K18 HR	405		
		1500 MG/K18 HR	406		
		1500 MG/K6 HR	413		
		1500 MG/K6 HR	414		
		1500 MG/K48 HR	423		
		2000 MG/K6 HR	501		
		2000 MG/K6 HR	503		
		2000 MG/K6 HR	513		
		2000 MG/K6 HR	514		
		2000 MG/K18 HR	518		
		2000 MG/K24 HR	520		
		2000 MG/K48 HR	522		

**Table 5:** Subset of significant and unique genes that distinguish between levels of centrilobular necrosis of the rat liver.

			Cluster Comparison	
Feature ID	Gene	Description	A vs B	

A_42_P464546	AI501407	TNFAIP3 interacting protein 2	1	2
A_42_P496622	AI232716	Similar to thioether S-methyltransferase	1	2
A_42_P552441	BI303289	Growth arrest specific 5	1	2
A_42_P565917	BF392498	Cytochrome P450, family 2, subfamily u, polypeptide 1	1	2
A_42_P681012	NM_013055	Mitogen activated protein kinase kinase kinase 12	1	2
A_42_P684538	NM_138827	Solute carrier family 2 (facilitated glucose transporter), member 1	1	2
A_42_P767698	BE097112	EBNA1 binding protein 2	1	2
A_42_P786624	NM_012693	Cytochrome P450, subfamily 2A, polypeptide 1	1	2
A_42_P788480	BF419374	Thyrotroph embryonic factor	1	2
A_43_P11472	NM_012580	Heme oxygenase (decycling) 1	1	2
A_43_P11681	NM_013048	Tocopherol (alpha) transfer protein	1	2
A_43_P12400	NM_024134	DNA-damage inducible transcript 3	1	2
A_43_P12595	NM_031576	P450 (cytochrome) oxidoreductase	1	2
A_43_P12996	NM_053955	Crystallin, mu	1	2
A_42_P487744	BF396233	Similar to 2410004L22Rik protein	1	3
A_42_P607568	AI176590	Similar to RIKEN cDNA C730048E16	1	3
A_42_P634040	AW918024	Ngg1 interacting factor 3-like 1 (S. pombe)	1	3
A_42_P634187	AW252746	Forkhead box D4	1	3
A_42_P677628	NM_031642	Core promoter element binding protein	1	3
A_42_P681533	AI237597	Transcribed locus, moderately similar to NP_034610.1 heat shock protein 1, alpha [Mus musculus]	1	3
A_43_P11142	Y10056	S100 calcium binding protein A11 (calizzarin) (predicted)	1	3
A_43_P11477	NM_012591	Interferon regulatory factor 1	1	3
A_43_P12806	NM_053439	RAN, member RAS oncogene family	1	3
A_43_P14163	NM_012615	Ornithine decarboxylase 1	1	3
A_43_P14782	AI406490	Tyrosine kinase, non-receptor, 2	1	3
A_43_P16550	CA509226	Splicing factor 3a, subunit 1 (predicted)	1	3
A_43_P19988	CB545293	Similar to CGI-94 protein (predicted)	1	3
A_42_P539275	AA891212	Replication factor C (activator 1) 3	2	3
A_42_P660046	BF551617	Kinesin family member 16B (predicted)	2	3
A_42_P780457	AI071307	Ectodermal-neural cortex 1	2	3
A_43_P11285	BQ209715	Similar to Cc2-27 (predicted)	2	3
A_43_P20438	CB545761	Small optic lobes homolog (Drosophila) (predicted)	2	3
