# Supplementary material for: Simultaneous clustering of gene expression data with clinical chemistry and pathological evaluations reveals phenotypic prototypes
Source: BMC Syst Biol. 2007 Feb 23;1:15. doi: 10.1186/1752-0509-1-15 (PMC1839893; doi:10.1186/1752-0509-1-15)
Supplement: Additional file 1 — Generation and clustering of simulated mixed data and real data with reduced dimensions of the microarray data. Supplemental_materials.pdf is a pdf file to be viewed with Adobe Acrobat. [file 1752-0509-1-15-S1.pdf]

Simulation of Data for Clustering using the Mod*k*-prototypes Algorithm

Numeric Data

A data set comprised of numeric data with 64 features and 33 objects was simulated from three distinct probability distributions. Normal deviates (mean 0 and standard deviation 20) were drawn at random from the 3 probability distributions generating 11 objects in each. Samples that belong to the same class are #s12-22, 33-43, and 44-54. The R code for generating the data was provided by Pablo Tamayo at the Broad Institute, Massachusetts Institute of Technology.

Categorical Data

A data set comprised of categorical data with 10 features and 33 objects was simulated from a hidden Markov model (HMM) using R code in the HMM discrete non-parametric package. The HMM contained 3 states modeled on levels of toxicity (no/low, moderate and severe) and 5 severity levels (none, minimal, mild, moderate and marked) of centrilobular necrosis observed in rat livers exposed to acetaminophen. An independent data set (cDNA microarray gene expression data from Heinloth et al. 2004) acquired from rat liver samples exposed to 50, 150, 1500 and 2000 mg/kg of APAP for 6, 24 and 48 hours was used to estimate transition probabilities from a set of ~700 differentially expressed genes then also a set of 700 genes selected at random. A third set of transition probabilities were manually created with a high probability ($p >= 0.6$) of visiting or remaining in the no/low toxicity state. A model of the HMM, a curve of the log-likelihood from the training, the transition and emission probabilities are below.

Figure S1

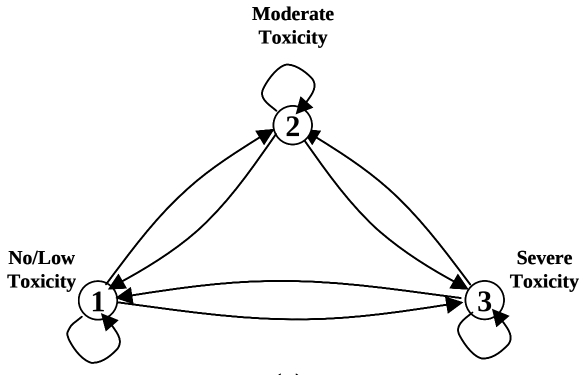

Figure S2

Log Likelihood of Model During Training

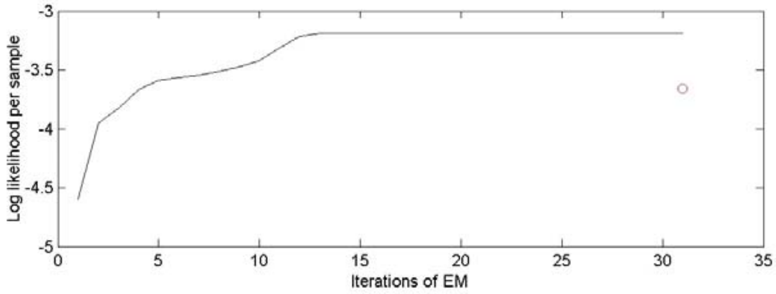

Transition Probabilities

	1	2	3
1	0.114	0.051	0.835
2	0.366	0.001	0.633
3	0.594	0.009	0.397

699 informative genes

	1	2	3
1	0.562	0.437	0.001
2	0.189	0.808	0.003
3	0.992	0.007	0.001

Randomly selected genes

	1	2	3
1	0.900	0.050	0.050
2	0.800	0.100	0.100
3	0.600	0.200	0.200

Manually created

Emission Probabilities

	Toxicity States		
Severity	No/low	Moderate	Severe
None	0.800	0.140	0.010
Minimal	0.100	0.240	0.040
Mild	0.050	0.240	0.050
Moderate	0.040	0.240	0.100
Marked	0.010	0.140	0.800

A mixed data set (sim_mixed_data.txt) was created from the merge of the numeric and the categorical simulated data sets. Clustering of the simulated mixed data was performed using the mod*k*-prototypes algorithm with adaptive weighting of the numeric and categorical data. After 50 trial clustering attempts over 2 to *k* possible clusters in the data, the mod*k*-prototypes algorithm partitioned the data into 3 groups with the appropriate samples into their respective class cluster (i.e., samples #s 12-22 together, 33-43 together and 44-54 together). The figure below illustrates the minimization of the DVI_CU index at *k* = 3.

Figure S3

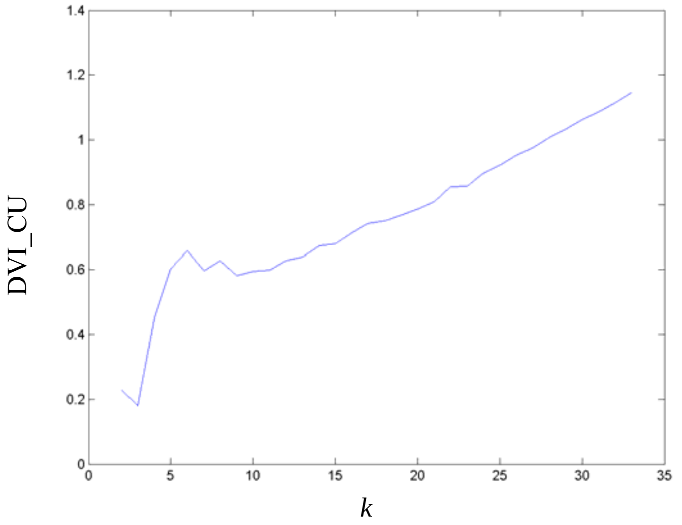

Reduction of Dimension of the Microarray Data for Mod*k*-prototypes Clustering with the Phenotypic Data

Principal Component Analysis (PCA) was performed on the ~3100 gene expression profiles obtained from the ANOVA model of the microarray data set comparing acetaminophen-treated samples with time-matched controls (see methods section). Of the 64 PCs obtained, the top 20 PCs (which differed in the range of the eigenvalues), the 10 clinical chemistry measurements and the 48 histopathology observations were used for clustering the samples. Using the mod*k*-prototypes algorithm with the weights set at

0.51, 0.24 and 0.25 for the α, β and γ respectively, $k = 3$ was determined to be the number of clusters in the data (see figure below). Furthermore, the assignment of samples to the clusters did not differ than when the ~3100 genes expression profiles were used with the phenotypic data to cluster the samples.

Figure S4

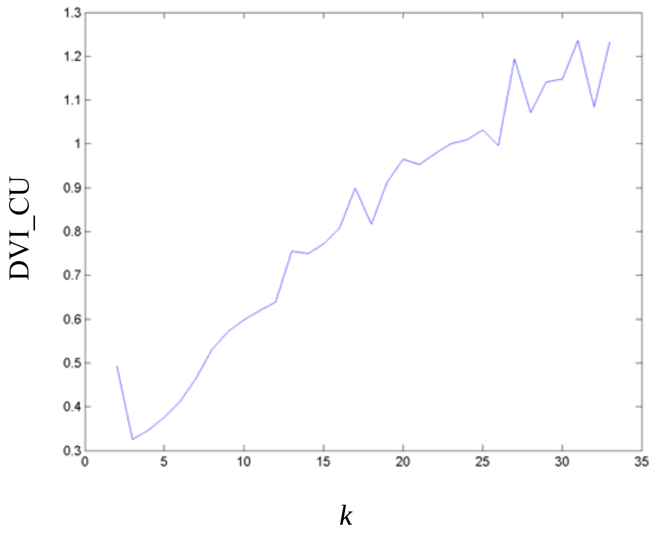

References

Heinloth AN, Irwin RD, Boorman GA, Nettesheim P, Fannin RD, Sieber SO, Snell ML, Tucker CJ, Li L, Travlos GS, Vansant G, Blackshear PE, Tennant RW, Cunningham ML, Paules RS. Gene expression profiling of rat livers reveals indicators of potential adverse effects. Toxicol Sci. 2004;80:193-202.
